# Comorbid Alzheimer's Disease and Type 2 Diabetes Microbiota Shape Age‐Associated Gut–Brain Axis Profiles

**DOI:** 10.1111/acel.70488

**Published:** 2026-04-21

**Authors:** Alessandro Atzeni, Jonas Mingaila, Gediminas Alzbutas, Rokas Lukoševičius, Justinas Drūteika, Kamila Łuczyńska, Rima Ramonaitė, Tadeusz Pietras, Kasper Sipowicz, Dovilė Kiudelytė, Karolina Keršytė, Emilija Keževičiūtė, Greta Najūtė, Juozas Kupčinskas, Jordi Mayneris‐Perxachs, Daiva Baltriukienė, Aurelijus Burokas

**Affiliations:** ^1^ Department of Biological Models, Institute of Biochemistry, Life Sciences Center Vilnius University Vilnius Lithuania; ^2^ Institute for Digestive Research Lithuanian University of Health Sciences Kaunas Lithuania; ^3^ Department of Neurology, Lithuanian University of Health Sciences Hospital of Lithuanian University of Health Sciences Kauno Klinikos Kaunas Lithuania; ^4^ Institute of Genetics and Animal Biotechnology of the Polish Academy of Sciences Magdalenka Poland; ^5^ The Second Department of Psychiatry, Institute of Psychiatry and Neurology in Warsaw Warsaw Poland; ^6^ Department of Clinical Pharmacology Medical University of Lodz Lodz Poland; ^7^ Department of Interdisciplinary Research in the Area of Social Inclusion The Maria Grzegorzewska University Warsaw Poland; ^8^ Dr. L. Kriaučeliūnas Small Animal Clinic, Faculty of Veterinary, Veterinary Academy Lithuanian University of Health Sciences Kaunas Lithuania; ^9^ Gastroenterology Department Lithuanian University of Health Sciences Kaunas Lithuania; ^10^ Integrative Systems Medicine and Biology Institut d'Investigació Biomèdica de Girona Dr. Josep Trueta (IDIBGI) Girona Spain

**Keywords:** aging, Alzheimer's disease, comorbidity, fecal microbiota transplantation, gut microbiota, gut–brain axis, type 2 diabetes mellitus

## Abstract

Alzheimer's disease (AD) and type 2 diabetes mellitus (T2DM) share metabolic and inflammatory mechanisms, potentially mediated by the gut microbiota, yet the neurobiological impact of comorbid AD+T2DM microbiota from elderly donors remains unexplored. Fecal microbiota from healthy, AD, T2DM, and AD+T2DM postmenopausal female donors (aged 56–89 years) was transplanted into antibiotic‐treated male mice. Behavioral testing, blood profiling, hippocampal neurotrophic gene expression, and 16S rRNA sequencing with taxonomic, functional, and metabolic analyses were performed. Human AD+T2DM microbiota displayed the greatest dysbiosis, characterized by enrichment of pro‐inflammatory taxa, depletion of butyrate‐producing genera, and loss of neuroprotective metabolic pathways. FMT induced robust engraftment, with AD+T2DM recipients diverging most from controls (PERMANOVA *R* ^2^ = 0.209, *p* = 0.001) and healthy recipients (PERMANOVA *R* ^2^ = 0.111, *p* = 0.002). Donor age contributed significantly to recipient microbiota variation (*R* ^2^ = 0.028, *p* = 0.006), suggesting transmission of aging‐associated microbial signatures. Hippocampal neurotrophic gene expression was most suppressed in AD+T2DM recipients (adjusted *p* value < 0.05) and negatively correlated with disease‐ and aging‐associated taxa and microbial functions (|r| > 0.4, FDR *p* < 0.05). AD recipients showed reduced olfactory discrimination and increased daytime locomotor activity. Metabolic network analysis revealed depletion of flavonoid, isoflavonoid, and lignan biosynthesis pathways in disease recipients. These findings suggest that microbiota from elderly donors with comorbid AD+T2DM may induce gut–brain axis alterations, linking aging, metabolic dysfunction, and neurodegeneration through convergent taxonomic, functional, and neurotrophic changes. We underscore the potential role of age‐associated gut microbial signatures in modulating neurobiological outcomes.

## Graphical abstract

Fecal microbiota transplantation from elderly donors with Alzheimer's disease, type 2 diabetes, or both conditions into antibiotic‐treated mice revealed that comorbid microbiota produce the most severe dysbiosis and strongest suppression of hippocampal neurotrophic gene expression. Disease‐derived microbiota were enriched in pro‐inflammatory taxa and depleted in butyrate producers, with specific genera negatively correlated with *Ngf*, *Igf*1, and *Creb*1 expression. These findings demonstrate that aged, diseased human microbiota drive gut–brain axis dysfunction through convergent taxonomic and functional alterations.

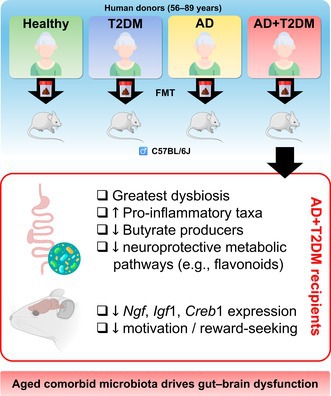

## 1 Introduction

Aging is the major risk factor for most chronic diseases, including neurodegenerative and metabolic disorders, reflecting the cumulative effects of molecular and cellular decline over time (Franceschi et al. 2018). Alzheimer's disease (AD) is a progressive neurodegenerative disorder and the leading cause of dementia worldwide (Safiri et al. 2024). Its neuropathology is characterized by extracellular deposition of amyloid‐β (Aβ) plaques, intracellular neurofibrillary tangles composed of hyperphosphorylated tau, synaptic dysfunction, and neuronal loss (DeTure and Dickson 2019). Despite intensive research, the etiology of AD remains multifactorial, encompassing genetic, metabolic, and environmental influences (Breijyeh and Karaman 2020). While genetic variants in APOE, APP, PSEN1, and PSEN2 influence susceptibility, the underlying biology of aging, particularly age‐associated molecular and cellular dysregulation, substantially shapes disease onset and progression (Breijyeh and Karaman 2020). Brain insulin resistance and neuroinflammation have emerged as pivotal mechanisms linking metabolic dysregulation to cognitive decline (Talbot et al. 2012), illustrating how fundamental aging processes intersect with disease phenotypes.

Type 2 diabetes mellitus (T2DM) is a chronic metabolic disorder characterized by insulin resistance, impaired insulin secretion, and hyperglycemia (Chatterjee and Mudher 2018). Epidemiological and mechanistic studies indicate that T2DM significantly increases the risk of developing AD (Vagelatos and Eslick 2013). Insulin signaling in the central nervous system regulates neuronal survival, synaptic plasticity, and neurotransmitter release (de la Monte and Wands 2008). Impairment of this pathway, especially through disruption of the insulin receptor–phosphoinositide 3‐kinase (PI3K)–protein kinase B (Akt), leads to reduced glucose utilization and impaired neurotrophic signaling (de la Monte and Wands 2008). Consequently, individuals with T2DM frequently exhibit cognitive deficits and structural brain alterations resembling early AD (Biessels and Despa 2018; Chatterjee and Mudher 2018). The bidirectional interaction between insulin resistance and Aβ pathology has led to the concept of AD as “type 3 diabetes,” reflecting the shared molecular features of impaired insulin signaling, mitochondrial dysfunction, and chronic inflammation in both disorders (Steen et al. 2005), and underscoring how aging‐related decline in metabolic and cellular resilience amplifies disease risk (Franceschi et al. 2018).

Recent advances implicate the gut microbiota as a critical mediator of these neuro–metabolic interactions (Cryan et al. 2020), with dysbiosis potentially accelerating hallmarks of aging such as chronic inflammation, impaired metabolic regulation, and loss of cellular homeostasis (Franceschi et al. 2018). The microbiota–gut–brain axis encompasses neural, immune, and endocrine pathways through which the intestinal microbiota influences brain function (Burokas et al. 2015). Microbial metabolites, including short‐chain fatty acids (SCFA), bile acids, and trimethylamine‐N‐oxide, modulate host glucose metabolism, lipid homeostasis, and neuroinflammation (Agus et al. 2021; Kunevičius et al. 2025). Dysbiosis, defined as an imbalance between protective and pro‐inflammatory taxa, increases intestinal and blood–brain barrier permeability, facilitating systemic exposure to microbial endotoxins such as lipopolysaccharides (LPS) (Zhuang et al. 2018). These molecules activate microglia and astrocytes, promoting cytokine release and Aβ aggregation (Ortiz‐Samur et al. 2025; Vijaya et al. 2024; Y. Zhao et al. 2015). Alterations in microbial diversity and composition have been documented in both AD and T2DM (Cryan et al. 2020; Megur et al. 2020, 2022; Zhuang et al. 2018), with overlapping enrichment of pro‐inflammatory genera and depletion of beneficial taxa (Zhang et al. 2022). Experimental evidence supports a causal role of the gut microbiota in modulating both cognitive and metabolic phenotypes (M.‐S. Kim et al. 2020). Fecal microbiota transplantation (FMT) from patients with AD or T2DM into germ‐free or antibiotic‐treated mice induces cognitive impairments, neuroinflammatory activation, and metabolic disturbances resembling donor pathology, whereas FMT from healthy donors can ameliorate these abnormalities (Arnoriaga‐Rodríguez et al. 2020, 2021; N. Kim et al. 2021; Mayneris‐Perxachs et al. 2022). Despite growing evidence linking gut microbiota alterations to both AD and T2DM, the combined impact of microbiota from individuals with comorbid AD and T2DM, conditions highly relevant to aging populations and geroscience research, remains unexplored. To date, no studies have directly assessed the effects of FMT from donors with concurrent AD and T2DM, highlighting a critical knowledge gap in understanding how aging‐related molecular and cellular alterations interact with gut microbial communities to influence neuro–metabolic health.

This study aimed to investigate how human‐derived microbiota from donors with AD, T2DM, or both diseases modulate aging‐relevant pathways affecting cognition, neurotrophic signaling, immune responses, and gut microbial ecology in recipient mice. Using integrated behavioral testing, hippocampal gene expression profiling, and 16S rRNA sequencing coupled with metabolic network analysis, we aimed to identify microbiota‐mediated mechanisms that intersect with hallmarks of aging to drive neurodegenerative and metabolic phenotypes. We hypothesized that fecal microbiota from individuals with comorbid AD and T2DM would produce the most pronounced alterations in recipient mice, reflected by disrupted neurotrophic gene expression, altered microbial composition, and impaired behavioral performance, thereby revealing how dual metabolic and neurodegenerative microbiota signatures synergistically influence age‐associated gut–brain axis dysfunction.

## 2 Methods

### 2.1 Study Design

Recipient mice underwent antibiotic‐mediated gut depletion followed by FMT from healthy, AD, T2DM, or AD+T2DM human donors. To explore human‐derived gut microbiota influence on physiological and neurological outcomes, recipient mice were tested for behavioral performances, metabolic and gastrointestinal functions, systemic inflammation, hippocampal neurotrophic gene expression, alongside characterization of gut microbiota composition and predicted functional profiles. An antibiotic‐treated group of mice exposed to sham saline transfer was used as a procedural control to confirm microbiota depletion and FMT engraftment, while mice receiving microbiota from healthy donors served as the biological reference group for comparisons with disease‐derived microbiota.

### 2.2 Participants Recruitment, Clinical Characterization, and Sample Collection

A total of 33 women aged 56–89 years were recruited from the Lithuanian University of Health Sciences (Kaunas, Lithuania), the Institute of Psychiatry and Neurology (Warsaw, Poland), and the Norbert Barlicki Teaching Hospital No. 1 of Medical University of Lodz (Lodz, Poland). Recruitment was conducted through clinical referrals and direct invitations by neurologists at the Neurology Outpatient Department of the Hospital of Lithuanian University of Health Sciences Kauno Klinikos (Kaunas, Lithuania). Participants were classified into four groups: (i) healthy individuals without cognitive impairments or a diagnosis of T2DM (healthy group); (ii) individuals with a confirmed diagnosis of T2DM but no AD or other forms of dementia (T2DM group); (iii) individuals with a confirmed diagnosis of AD but without T2DM (AD group); (iv) individuals with a confirmed diagnosis of AD and T2DM mellitus (AD+T2DM group). The groups were age‐matched. AD was diagnosed according to the National Institute of Neurological and Communicative Disorders and Stroke–Alzheimer's Disease and Related Disorders Association (NINCDS‐ADRDA) criteria (McKhann et al. 1984), while T2DM was confirmed based on the American Diabetes Association (ADA) diagnostic criteria (Gabir et al. 2000). Cognitive status was assessed by a clinical psychologist using the Mini‐Mental State Examination (MMSE) (Folstein et al. 1975) and additional neuropsychological tests, and daily functioning and behavioral changes were evaluated using the Blessed Dementia Scale (BDS) (Blessed et al. 1968). Participants were excluded if they had received antibiotics, probiotics, glucocorticoids, non‐steroidal anti‐inflammatory drugs, alcohol, or narcotic substances, or had experienced gastrointestinal infections within the previous four weeks. Additional exclusion criteria included abnormal brain imaging (CT or MRI) indicative of non‐degenerative pathology (e.g., ischemic foci, tumors, hydrocephalus, or post‐traumatic lesions); severe somatic illness (e.g., decompensated cardiac, renal, or hepatic insufficiency); a history of malignancy treated within the past ten years; chronic gastrointestinal disease or abdominal surgery; and severe dementia (MMSE < 11 points, Blessed Dementia Rating Scale > 22 points). All procedures involving human participants were conducted in accordance with the Declaration of Helsinki and were approved by the Kaunas Regional Biomedical Research Ethics Committee at the Lithuanian University of Health Sciences (Approval No. BE‐2‐55) and the Bioethics Committee at the Institute of Psychiatry and Neurology in Warsaw (Approval No. 23/2022). Stool samples were collected from donors who provided written informed consent and were stored at −80°C. Subsequently, the samples were processed for FMT preparation as described in the Supporting Information Methods.

### 2.3 Animals and Fecal Microbiota Transplantation Experimental Procedures

Animal procedures were conducted in strict accordance with the guidelines of the European Communities Council Directive 2010/63/EU and approved by the Lithuania State Food and Veterinary Service, Animal Ethics Experimentation Committee (No. G2‐239). The study involved 68 male C57BL/6J mice, aged 8 weeks at the beginning of the study. Animals were housed under standard conditions: a 12‐h light/dark cycle (light on at 7 AM and off at 7 PM), temperature maintained at 22°C ± 1°C, humidity at 50%–60%, with autoclaved food and water available ad libitum. Mice were housed (2 per cage) in 36 × 15 cm cages covered with filters. Bedding and enrichment items within the cages were also autoclaved. Mice were randomly divided into five experimental groups, as they were comparable in condition and body size, with average group weights differing by no more than 1.5 g: healthy group (*n* = 15), received microbiota from healthy individuals; T2DM group (*n* = 17), received microbiota from patients with T2DM; AD group (*n* = 20), received microbiota from AD patients; AD+T2DM group (*n* = 10), received microbiota from patients with both AD and T2DM. An antibiotic‐treated control group (*N* = 6) was included to evaluate microbiota depletion and the efficacy of FMT engraftment. These mice received a sham transfer consisting of sterile saline following the same gavage procedure used for FMT recipients. This control is essential to distinguish effects attributable to donor microbiota from those resulting from antibiotic‐induced depletion, recolonization dynamics, or procedural aspects of the transplantation technique (Secombe et al. 2021). FMT samples were prepared as previously described (Mingaila et al. 2023), by weighing 1.1–1.3 g of fresh fecal material into a sterile 15 mL tube. For each gram of fecal material, 10 mL of phosphate‐buffered saline (PBS) containing 15% glycerol, or alternatively, saline with 15% glycerol, was added. The mixture was first vigorously shaken by hand and then placed on a shaker at maximum speed until a homogeneous suspension was achieved, typically within 5 min. The suspension was subsequently centrifuged at 2000 rpm for 5 min, and the resulting supernatant was collected and stored at −80°C until use. Each human fecal microbiota sample was transplanted into a single mouse cage housing two animals, resulting in a 1:2 donor‐to‐recipient ratio. No pooling of fecal samples across human donors was performed. This ensured that each pair of recipient mice received microbiota from an individual, unique donor. To reduce the natural gut microbiota, mice received a combination of antibiotics and antifungal agents prior to FMT, as previously described (Mingaila et al. 2023). The treatment included ampicillin (1 g/L), metronidazole (1 g/L), vancomycin (500 mg/L), ciprofloxacin (200 mg/L), imipenem (250 mg/L), and fluconazole (1 g/L). These agents were mixed and administered once daily for 14 days via oral gavage (0.2 mL per mouse, 18 ga × 30 mm). Gastrointestinal cleansing was performed one day after the antibiotic regimen. Following a 12‐h fast, mice received a bowel‐cleansing solution (macrogol and saline) via gastric gavage (18 ga × 30 mm). After a 4‐h interval post‐cleanse, mice were anesthetized using 2% isoflurane gas, and a donor microbiota solution was administered intrarectally. Since the microbiota suspension was liquid, it was able to reach the cecum. This intrarectal FMT was performed once only, at the start of the experiment. Subsequently, starting three days after the intrarectal procedure, mice received oral FMT twice weekly via gavage (18 ga × 30 mm, 0.2 mL per mouse) for a total duration of 17 weeks, following a protocol adapted from previous studies (Arnoriaga‐Rodríguez et al. 2020). The 17‐week duration was selected to sustain the effect of the transferred microbiota for approximately three months, with behavioral and physiological assessments conducted thereafter. Animal weight and general health were monitored weekly throughout the experimental period to ensure welfare.

### 2.4 Behavioral Tests

Behavioral tests began nine weeks after the initial FMT procedure, with a minimum interval of two days between different tests. Prior to each test, mice were acclimated to the testing environment for 1 h. Test mazes were cleaned with distilled water between animals to eliminate residual odors and subsequently cleaned with 70% ethanol after each testing session. Behavioral assessments included the Open Field Test, Elevated Plus Maze, Novel Object Recognition, Y‐maze, Running Wheel, Social Maze, and Glucose Preference Test. These were used to evaluate locomotor activity, anxiety‐like behavior, cognition, sociability, and anhedonia. Detailed protocols are available in the Supporting Information.

### 2.5 Metabolic and Gastrointestinal Function Tests

In addition, mice underwent metabolic and gastrointestinal assessments, including the glucose tolerance test and the gastrointestinal transit time. Detailed descriptions of the procedures are provided in the Supporting Information.

### 2.6 Hematological and Immune Assays

To evaluate systemic inflammation and physiological status, mice were subjected to hematological and immune assays, including peripheral blood cell counts and plasma lipopolysaccharide (LPS) detection. Detailed descriptions of the procedures are provided in the Supporting Information.

### 2.7 Hippocampal Targeted Gene Expression Analysis

Targeted gene expression analysis was performed on mice hippocampal tissue. Quantitative PCR was performed to assess changes in gene expression of *Ngf* (encoding beta‐nerve growth factor), *Igf1* (encoding insulin‐like growth factor 1), *Fos* (Fos proto‐oncogene, AP‐1 transcription factor subunit), *Arc* (encoding activity‐regulated cytoskeleton‐associated protein), *Creb1* (encoding cyclic AMP‐responsive element‐binding protein 1), and *Dlg4* (Discs large MAGUK scaffold protein 4). These genes were selected for their established roles in synaptic plasticity, learning, memory, and neurotrophic signaling, key processes often disrupted in AD and metabolic disorders. Relative gene expression levels were calculated by normalizing the expression of genes of interest to that of *Gapdh* (Glyceraldehyde‐3‐phosphate dehydrogenase). Detailed descriptions of the procedures are provided in the Supporting Information.

### 2.8 16S rRNA Sequencing and Processing

Mice cecal contents were collected after animal culling into sterile 1.5 mL tubes. The samples were immediately placed on dry ice and stored at −80°C. Bacterial DNA was extracted from human and murine stool samples using the QIAamp Fast DNA Stool Mini Kit (QIAGEN, Hilden, Germany) following the manufacturer's instructions. The V3‐V4 region of the bacterial 16S rRNA gene was amplified using dual‐index primers following Fadrosh et al. (2014). Amplicon library preparation, quality control, normalization, pooling, and final quantification were performed using standard protocols and reagents. Libraries were sequenced on an Illumina MiSeq platform (Illumina Inc., San Diego, CA, USA) using a 600‐cycle paired‐end kit with a 10% control PhiX spike‐in. Complete procedural details, including PCR conditions, verification, normalization, and quantification, are available in Supporting Information Methods Section 6. Bioinformatic processing included demultiplexing with Pheniqs (Galanti et al. 2021) and primer removal and quality filtering using cutadapt (Martin 2011). Amplicon Sequence Variants (ASVs) were inferred using the DADA2 pipeline (Callahan et al. 2016), including error modeling, paired‐end merging, chimera removal, and length filtering. Taxonomy was assigned to the genus level using the SILVA reference database (Quast et al. 2012) (version 138.2). A detailed description of all steps is provided in the Supporting Information.

### 2.9 Statistical Analyses

Statistical analyses were conducted in R (v4.5.0). Data distributions were assessed for normality using the Shapiro–Wilk test. Depending on distribution, group differences were evaluated using one‐way ANOVA or the Kruskal–Wallis test. When omnibus tests were significant, post hoc pairwise comparisons were performed using Tukey's HSD or Dunn's test. To account for multiple testing, Benjamini‐Hochberg correction was applied to control the false discovery rate (FDR), and both raw and adjusted *p* values were reported. Effect sizes (estimates of group differences) were calculated and presented alongside *p* values to facilitate interpretation. Analyses were conducted separately for hippocampal targeted gene expression, blood count, behavioral, and physiological measures to account for the non‐independence of outcomes across domains. Comparisons were made between the Control and FMT groups, as well as among FMT groups. A threshold of *p* < 0.05 was considered statistically significant.

### 2.10 Microbiota Analysis

Taxonomic counts were aggregated at the genus level. Low‐prevalence and low‐abundance taxa were filtered out, retaining only those present in at least 10% of samples with a minimum of 10 reads. Alpha diversity was explored by calculating the Chao1, Shannon, and Simpson indices (Chao 1984; Shannon 1948; Simpson 1949). Differences in alpha diversity between experimental groups were evaluated using the Kruskal–Wallis test. For indices showing significant group differences, pairwise comparisons were conducted using Dunn's post hoc test with Benjamini–Hochberg correction to control for multiple testing. Only comparisons with an adjusted *p* < 0.05 were considered statistically significant.

Filtered genus‐level microbial counts were transformed using the centered log‐ratio (CLR) to account for compositionality (Aitchison 1982). Principal Component Analysis (PCA) was then performed on the CLR‐transformed counts, and the proportion of variance explained by the first two components (PC1 and PC2) was calculated. PCA scores were plotted with group‐specific colors and 95% confidence ellipses to visualize clustering patterns. The beta diversity was assessed using Euclidean distances on CLR‐transformed counts (Aitchison distance (Aitchison 1982)). Global differences on Aitchison distances among groups were tested using permutational multivariate analysis of variance (PERMANOVA), and pairwise PERMANOVA comparisons were performed to identify specific group differences, with *p* values corrected for multiple testing using the Benjamini‐Hochberg method. PICRUSt2 (Phylogenetic Investigation of Communities by Reconstruction of Unobserved States 2) (Douglas et al. 2019) was used to predict the functional composition of the microbial community. Representative sequences (as FASTA file) and corresponding ASV abundance (in BIOM format) were used as input for downstream PICRUSt2 prediction of metagenomic functional content. Functional profiling of the predicted gut microbiota was inferred from KEGG Orthologs (KO) abundances. KOs were then mapped to both Gut Brain Modules (GBM) and Gut Metabolic Modules (GMM) (Vieira‐Silva et al. 2016) computed from curated databases. Generated GBM and GMM abundance tables were filtered to include only those present in ≥ 10% of samples with at least 10 reads, CLR‐transformed, and used for downstream analyses. Differential abundance analysis was performed using the ANCOM‐BC (Analysis of Compositions of Microbiomes with Bias Correction) (Lin and Peddada 2020) framework to identify taxa and functional modules that differed significantly between experimental groups. Pairwise comparisons were conducted between study groups. For each comparison, ANCOM‐BC was run with the group variable as the predictor, using the Benjamini–Hochberg method to adjust *p* values. The first group alphabetically (or explicitly set reference) was used as the reference for log fold change interpretation. Taxa were considered significantly differentially abundant if they exhibited an absolute log fold change ≥ 1 and an adjusted *p* value < 0.05. Spearman Correlation was tested between differentially abundant gut microbiota features across FMT groups, host hippocampal gene expression markers and behavioral tests that showed significant group‐level differences. Gene expression data were log‐transformed with a small pseudocount to avoid zero values. Taxonomic abundances, functional modules, gene expression markers, and behavioral test scores were standardized or transformed as appropriate. Pairwise Spearman correlations were computed between (i) individual microbial taxa and hippocampal gene expression markers, (ii) functional modules and hippocampal gene expression markers, (iii) microbial taxa and behavioral measures, and (iv) functional modules and behavioral measures. Resulting correlation coefficients were compiled into matrices, and statistical significance was adjusted for multiple testing using the FDR method. Correlations exceeding |r| > 0.4 with FDR‐adjusted *p* values < 0.05 were considered significant.

### 2.11 Metabolic Network Analysis (MetNet)

To complement the ASV‐based taxonomic and functional analyses, a parallel metabolic network inference workflow was implemented using the same demultiplexed 16S rRNA sequencing data. Operational Taxonomic Units (OTUs) were generated through an independent clustering pipeline to enable reaction‐level feature extraction within QIIME 2 using the q2‐metnet plugin (version 1.0.2). The AGREDA metabolic model was applied at the genus level to compute reaction and subsystem flux scores, which were subsequently analyzed using generalized linear mixed‐effects models (glmmTMB) to identify group‐dependent alterations in microbial metabolic network structure. Full methodological details, including OTU clustering, taxonomic assignment, feature generation, and modeling parameters, are provided in the Supporting Information.

## 3 Results

### 3.1 General Characteristics and Gut Microbiota Profiles of Human Donors

Human donors did not significantly differ among groups in age, with all donors falling in a comparable older adult range, nor in demographic distribution (Table S1).

**FIGURE 1:**
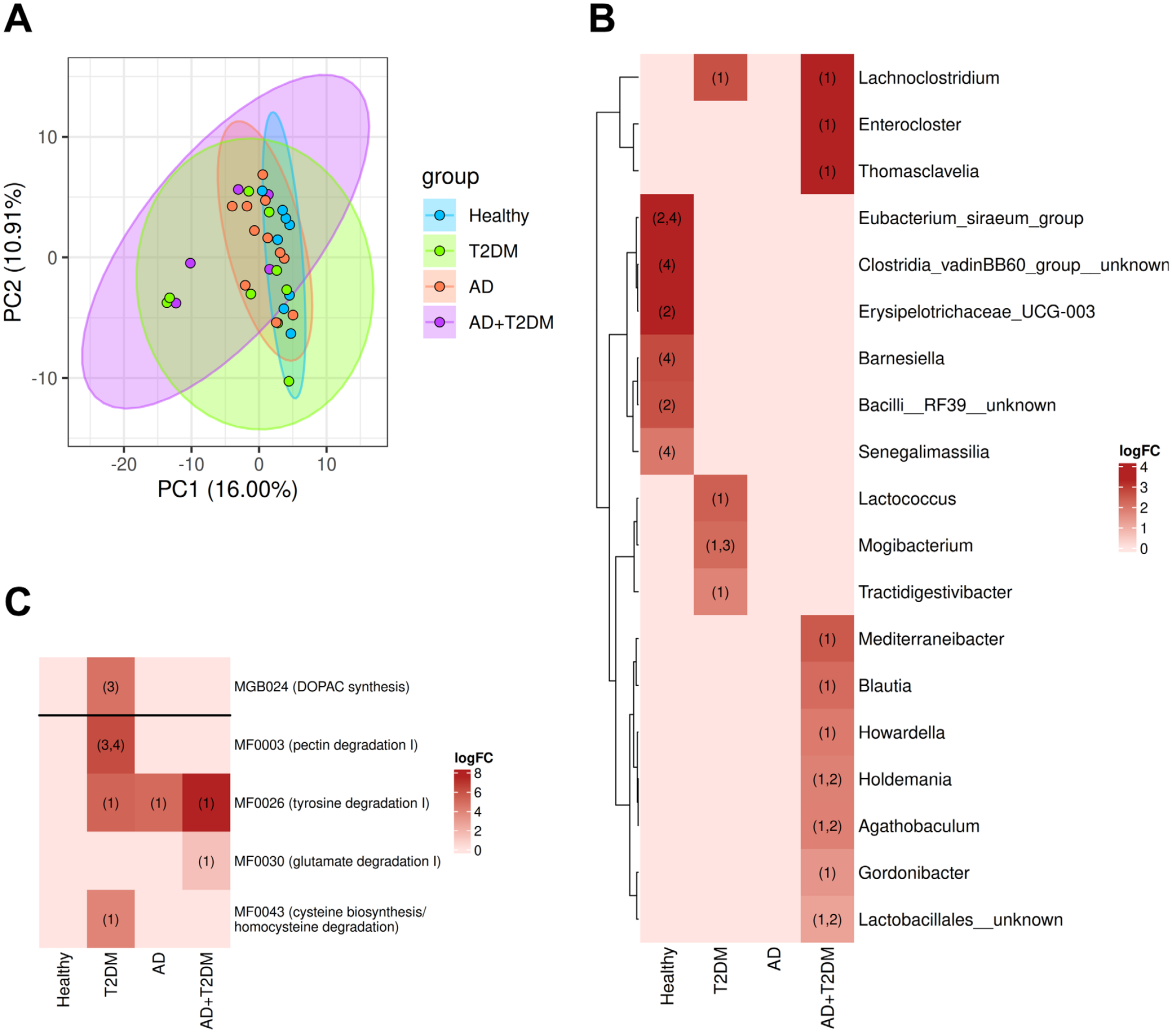
(A) Principal component analysis plot of human donors' gut microbial community. Each point represents an individual sample, colored according to the study group (Healthy, T2DM, AD, or AD+T2DM). Ellipses represent 95% confidence intervals, highlighting clustering patterns between disease and control groups. Axes indicate the proportion of variance explained by PC1 and PC2. (B) Heatmap of differentially abundant human microbiota taxa across study groups. (C) Heatmap of differentially abundant human microbiota functional modules across study groups. Each cell displays the LogFC of a feature abundance, indicating the magnitude and direction of differential abundance between study groups. Only taxa with LogFC > ±1 and adjusted *p* value < 0.05 are shown. Numbers within cells denote the comparison(s) where the feature is significantly enriched in the indicated group: (1) versus Healthy, (2) versus T2DM, (3) versus AD, (4) versus AD+T2DM.

There were no differences between study groups in alpha diversity indices calculated (Table S2). The PCA among human donor groups showed that the first two principal components (PC1 and PC2) explained respectively 16.0% and 10.9% of the variance in microbial community structure. Distinct clustering patterns were observed, with partial overlap between groups (Figure 1A).

Global PERMANOVA test for any group difference revealed that group membership explained a small but statistically significant portion of the variation (*R* ^2^ = 0.122, adjusted *p* = 0.032) in the microbial community composition. In contrast, neither donor age nor donor nationality had a significant effect on overall microbial structure. Residual 82.4% indicates that the majority of variation was attributable to inter‐individual differences not captured by these factors (Table S3). Pairwise PERMANOVA analysis at the genus level revealed that microbial community composition differed significantly between healthy donors and both AD+T2DM (*R* ^2^ = 0.151, adjusted *p* = 0.005) and T2DM (*R* ^2^ = 0.099, adjusted *p* = 0.031) groups. In contrast, no significant differences were observed between other groups (Table 1).

**TABLE 1:** Pairwise PERMANOVA analysis of gut microbiota composition in human donors and experimental mice groups.

Comparison	Df	Sum of squares	*R* ^2^	*F*	*p* adj.
Human donors					
AD+T2DM versus AD	1	1128.7	0.0803	1.222	0.159
AD+T2DM versus healthy	1	1722.2	0.1507	1.951	0.005
AD+T2DM versus T2DM	1	839.3	0.0650	0.834	0.577
AD versus healthy	1	867.6	0.0554	0.996	0.449
AD versus T2DM	1	982.5	0.0541	1.030	0.399
Healthy versus T2DM	1	1540.3	0.0994	1.656	0.031
Mice: control vs FMT recipients					
Control versus AD	1	684.4	0.1199	3.268	0.001
Control versus T2DM	1	626.4	0.1345	3.265	0.001
Control versus AD+T2DM	1	640.1	0.2091	3.700	0.001
Control versus healthy	1	558.1	0.1292	2.818	0.003
Mice: among FMT recipients					
AD versus T2DM	1	394.0	0.0513	1.894	0.006
AD versus AD+T2DM	1	435.2	0.0711	2.143	0.005
AD versus healthy	1	520.7	0.0691	2.450	0.001
T2DM versus AD+T2DM	1	431.7	0.0844	2.306	0.003
T2DM versus healthy	1	435.7	0.0676	2.176	0.006
AD+T2DM versus healthy	1	549.6	0.1107	2.863	0.002

*Note:* PERMANOVA was performed using Aitchison distance on centered log‐ratio–transformed genus‐level data. Effect size is reported as *R* ^2^, and *p* values were FDR‐corrected using the Benjamini–Hochberg method. Significant *p* < 0.05.

Differential abundance analysis of the human gut microbiota revealed disease‐specific microbial signatures (Figure 1B). Several taxa were enriched in disease groups relative to healthy donors, including *Lachnoclostridium*, *Holdemania*, *Agathobaculum*, and unknown *Lactobacillales* genus (enriched in T2DM and AD+T2DM), *Enterocloster*, *Thomasclavelia*, *Mediterraneibacter*, *Blautia*, *Howardella*, and *Gordonibacter* (enriched in AD+T2DM), *Lactococcus* and *Tractidigestivibacter* (enriched in T2DM). Conversely, taxa enriched in healthy donors included *Eubacterium siraeum* group (vs. both T2DM and AD+T2DM), *Clostridia* vadinBB60 group unknown genus, *Barnesiella*, and *Senigalimassilia* (vs. AD+T2DM), *Erysipelotrichaceae* UCG‐003 and an unknown *Bacilli* RF39 genus (vs. T2DM). Finally, *Mogibacterium* was enriched in T2DM relative to both healthy and AD donors.

Differential abundance analysis of predicted microbial functional modules revealed disease‐specific metabolic shifts (Figure 1C). T2DM donors showed enrichment of DOPAC synthesis GBM (vs. AD), pectin degradation I (vs. AD and healthy), and cysteine biosynthesis/homocysteine degradation GMM (vs. T2DM and healthy). AD+T2DM donors were characterized by enrichment of glutamate degradation I GMM (vs. healthy). In contrast, healthy individuals exhibited reduced tyrosine degradation I GMM compared with all disease groups.

### 3.2 Microbiota Engraftment and Host Responses to FMT

Antibiotic‐treated mice receiving sham saline transfer were compared with FMT recipients to evaluate microbiota engraftment and associated host physiological and behavioral responses.

Significant differences in calculated alpha diversity indices were observed between the control and the FMT groups (Table S4). Both the Shannon and Simpson indices were significantly higher in the AD group compared to the control group (adjusted *p* = 0.022; adjusted *p* = 0.013, respectively). The Shannon index was also significantly higher in the AD+T2DM group compared to the control group (adjusted *p* = 0.022).

**FIGURE 2:**
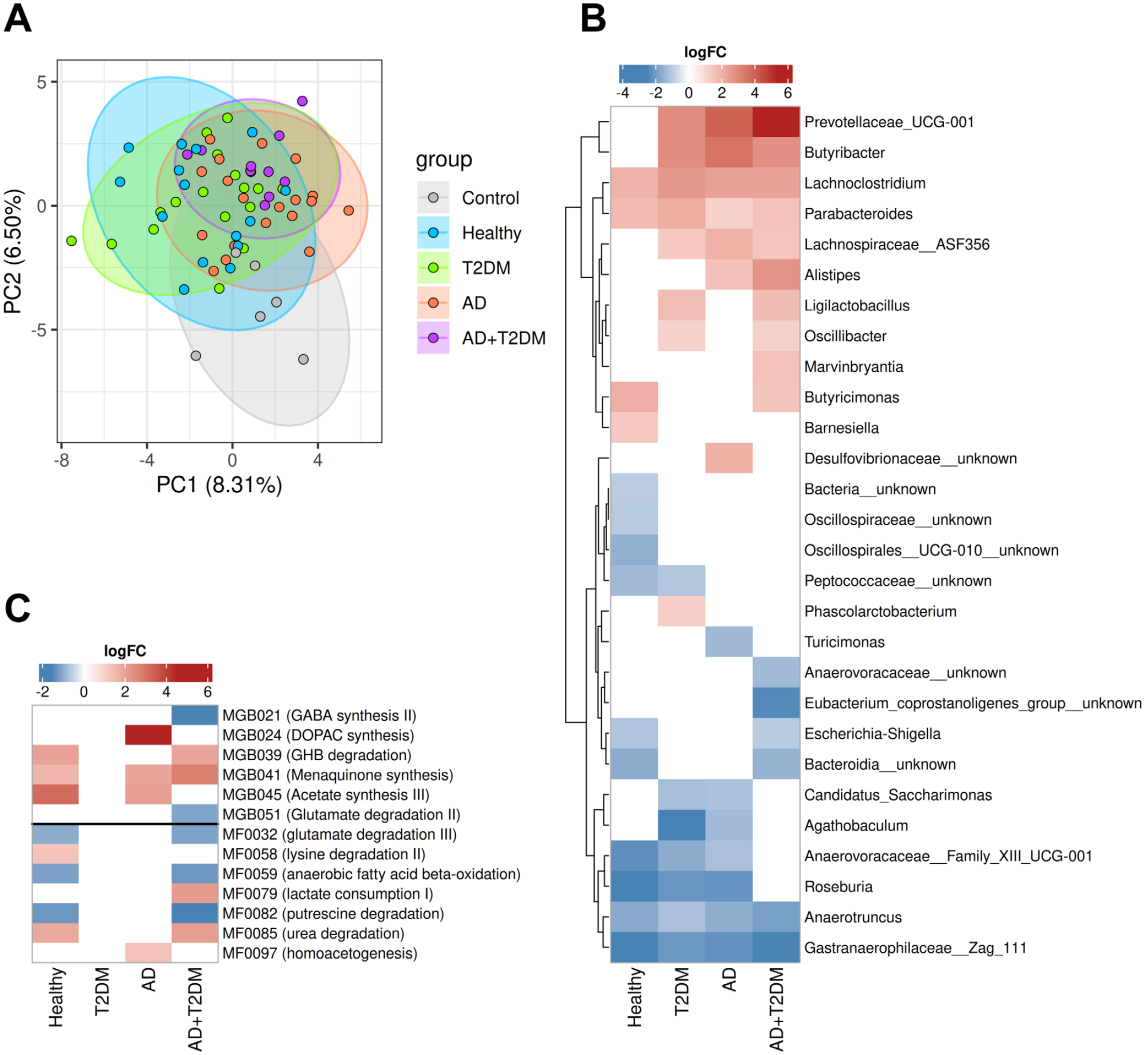
(A) Principal component analysis plot of mice gut microbial community. Each point represents an individual sample, colored according to the study group (Control, Healthy, T2DM, AD, or AD+T2DM). Ellipses represent 95% confidence intervals, highlighting clustering patterns between disease and control groups. Axes indicate the proportion of variance explained by PC1 and PC2. (B) Differentially abundant taxa enriched in the control group compared to FMT groups. (C) Differentially abundant functional modules enriched in the control group compared to FMT groups. The heatmaps show features with LogFC > ±1 and adjusted *p* value < 0.05. Each cell represents the LogFC value for a feature, where negative values (blue) indicate enrichment in the control group, and positive values (red) indicate enrichment in the comparison group. Color intensity reflects the magnitude of differential abundance.

The PCA plot (Figure 2A) showed that the first two principal components (PC1 and PC2) explained 8.3% and 6.5% of the variance, respectively. FMT samples tended to cluster separately from controls, indicating a shift in microbial community structure following transplantation.

The global PERMANOVA results indicate that microbiota composition differed significantly between the control and the FMT groups (adjusted *p* = 0.001). The model explained approximately 10.5% of the total variation, while most of the variation (89.5%) remained within groups (Table S5). Despite this high within‐group variability, the significant *p* value demonstrates that the gut microbial communities were altered by FMT treatment compared to controls, supporting the PCA observations of partial separation between groups.

Pairwise PERMANOVA comparisons showed significant differences in microbiota composition between the control group and the FMT groups (Table 1). Compared with controls, distinct microbial shifts were observed in the AD, T2DM, and AD+T2DM groups (all adjusted *p* = 0.001), as well as in the healthy FMT group (adjusted *p* = 0.003). Notably, the AD+T2DM FMT group showed the highest explained variance (*R* ^2^ = 0.209), suggesting that donor disease status had a marked influence on the gut microbiota established in recipient mice.

Differential abundance analysis revealed distinct microbial signatures between control and FMT groups (Figure 2B). Several genera were enriched in FMT recipients relative to controls, including *Prevotellaceae* UCG‐001, *Butyribacter* and *Lachnospiraceae* ASF365 (enriched in T2DM, AD, AD+T2DM), *Lachnoclostridium* and *Parabacteroides* (enriched in all FMT groups), *Alistipes* (enriched in AD and AD+T2DM). Additional donor‐specific enrichments were observed, such as *Ligilactobacillus* and *Oscillibacter* (enriched in T2DM and AD+T2DM), *Marvinbryantia* (enriched in AD+T2DM), *Butyricimonas* (enriched in healthy and AD+T2DM), and *Barnesiella* (enriched in healthy). In contrast, several taxa were enriched in controls, including *Oscillospiraceae* unknown genus and *Oscillospirales* UCG‐001 unknown genus (vs. healthy), *Peptococcaceae* unknown genera, *Escherichia‐Shigella* and *Bacteroidia* unknown genera (vs. healthy and T2DM). *Turicimonas* (vs. AD), *Anaerovoraceae* and *Eubacterium coprostanoligenes* group unknown genus (vs. AD+T2DM), *Candidatus Saccharimonas* and *Agathobaculum* (vs. T2DM and AD), *Anaerovoraceae* Family XIII UCG‐001 and *Roseburia* (vs. healthy, T2DM, and AD), *Anaerotruncus* and *Gastranaerophilaceae* Zag‐111 (vs. all FMT groups).

Differential abundance analysis of functional modules showed that FMT recipients exhibited enrichment of several GMB including DOPAC synthesis (enriched in AD), acetate synthesis III (enriched in AD and healthy), γ‐hydroxybutyric acid degradation (enriched in healthy and AD+T2DM), and menaquinone synthesis (enriched in healthy, AD, and AD+T2DM). In contrast, GABA synthesis II was enriched in control versus AD+T2DM. Among GMM, FMT groups showed enrichment in lysine degradation (enriched in healthy), lactate consumption (enriched in AD+T2DM), urea degradation (enriched in healthy and AD+T2DM), and homoacetogenesis (enriched in AD). Conversely, controls were enriched in glutamate degradation III, anaerobic fatty acid beta‐oxidation, putrescine degradation (vs. healthy and AD+T2DM), and glutamate degradation II (vs. AD+T2DM) (Figure 2C).

Omnibus tests revealed significant differences between antibiotic‐treated control mice and FMT recipients across multiple domains (Table S6). Specifically, the expression of all tested neurotrophic and synaptic plasticity–related genes differed significantly between control and FMT groups. LYM, WBC, and GRAN blood counts, daytime locomotor activity, sniffing discrimination index, and cecum weight were also significantly affected.

Pairwise comparisons indicated that control mice exhibited higher *Creb1* expression and circulating immune cell counts (LYM, WBC) relative to selected FMT groups. In contrast, FMT recipients, particularly AD mice, displayed increased daytime locomotor activity and greater cecum weight. Sniffing test discrimination index was reduced in AD recipients compared with controls (Tables 2 and S7).

**TABLE 2:** Significant pairwise differences in hippocampal gene expression markers, blood count, and behavioral measures among experimental mice groups.

Variable	(1) control	(2) healthy	(3) T2DM	(4) AD	(5) AD+T2DM
*Ngf* (relative gene exp.)	0.0006 [0.0002]	0.0004 [0.0001]^c^, ^d^	0.0007 [0.0003]^b^, ^e^	0.0007 [0.0003]^b^, ^e^	0.0005 [0.0002]^c^, ^d^
*Igf1* (relative gene exp.)	0.0001 [0.0002]	5.0 × 10^−5^ [3.0 × 10^−5^]^c^, ^d^	0.0001 [9.0 × 10^−5^]^b^, ^e^	0.0001 [5.0 × 10^−5^]^b^, ^e^	6.0 × 10^−5^ [5.0 × 10^−5^]^c^, ^d^
*Fos* (relative gene exp.)	0.0020 [0.0016]	0.0011 [0.0010]^d^	0.0020 [0.0018]	0.0026 [0.0009]^b^, ^e^	0.0014 [0.0009]^d^
*Arc* (relative gene exp.)	0.0380 [0.0207]	0.0292 [0.0111]^c^, ^d^	0.0397 [0.0277]^b^, ^e^	0.0496 [0.0069]^b^, ^e^	0.0241 [0.0118]^c^, ^d^
*Creb1* (relative gene exp.)	0.0086 ± 0.0026^b^, ^d^	0.0043 ± 0.0017^a^, ^c^, ^d^	0.0099 ± 0.0031^b^, ^e^	0.0104 ± 0.0019^a^, ^b^, ^e^	0.0051 ± 0.0016^c^, ^d^
LYM (10^9^/L)	20.2 [18.7]^c^	18.7 [3.2]	12.3 [5.4]^a^	16.7 [2.4]	16.4 [1.3]
WBC (10^9^/L)	25.6 [4.2]^c^, ^e^	24.3 [5.1]^c^, ^e^	21.1 [6.2]^a^, ^b^, ^d^	23.3 [4.2]^c^, ^e^	20.6 [1.4]^a^, ^b^, ^d^
GRAN (10^9^/L)	5.0 [1.9]	5.4 [1.3]^c^, ^e^	4.0 [1.0]^b^, ^d^	5.2 [1.4]^c^, ^e^	3.8 [0.9]^b^, ^d^
Running wheel daytime (12 h) (rev)	782.5 [541.5]^d^	768.0 [319.5]	1979.0 [1704.0]	3599.5 [2904.2]^a^	956.5 [1496.2]
Sniffing test DI	0.7 [0.1]^d^	0.7 [0.3]^d^	0.5 [0.4]^d^	0.3 [0.3]^a^, ^b^, ^c^, ^e^	0.6 [0.1]^d^
Cecum weight (g)	0.6 [0.1]^b^, ^c^, ^d^	0.8 [0.2]^a^, ^d^	0.8 [0.3]^a^	0.9 [0.2]^a^	0.8 [0.1]^d^

*Note:* Data are presented as mean ± SD or median [IQR]. Post hoc pairwise comparisons were performed only when the omnibus test was significant, using Tukey's HSD and Dunn's test, as appropriate. Significant differences (Benjamini–Hochberg adjusted *p* < 0.05) are indicated by superscripts.

Abbreviations: DI, discrimination index; GRAN, granulocytes; LYM, lymphocytes; WBC, white blood cells.

^a^ Vs. control.

^b^ Vs. healthy.

^c^ Vs. T2DM.

^d^ Vs. AD.

^e^ Vs. AD+T2DM.

### 3.3 FMT Donor‐Dependent Gut Microbiota Profiles and Impact on Host Physiology and Behavior

To evaluate the biological effects of disease‐associated microbiota, comparisons primarily focused on mice receiving microbiota from healthy donors versus those receiving microbiota from AD, T2DM, or AD+T2DM donors.

There were no differences in calculated alpha diversity indices across FMT groups (Table S8).

**FIGURE 3:**
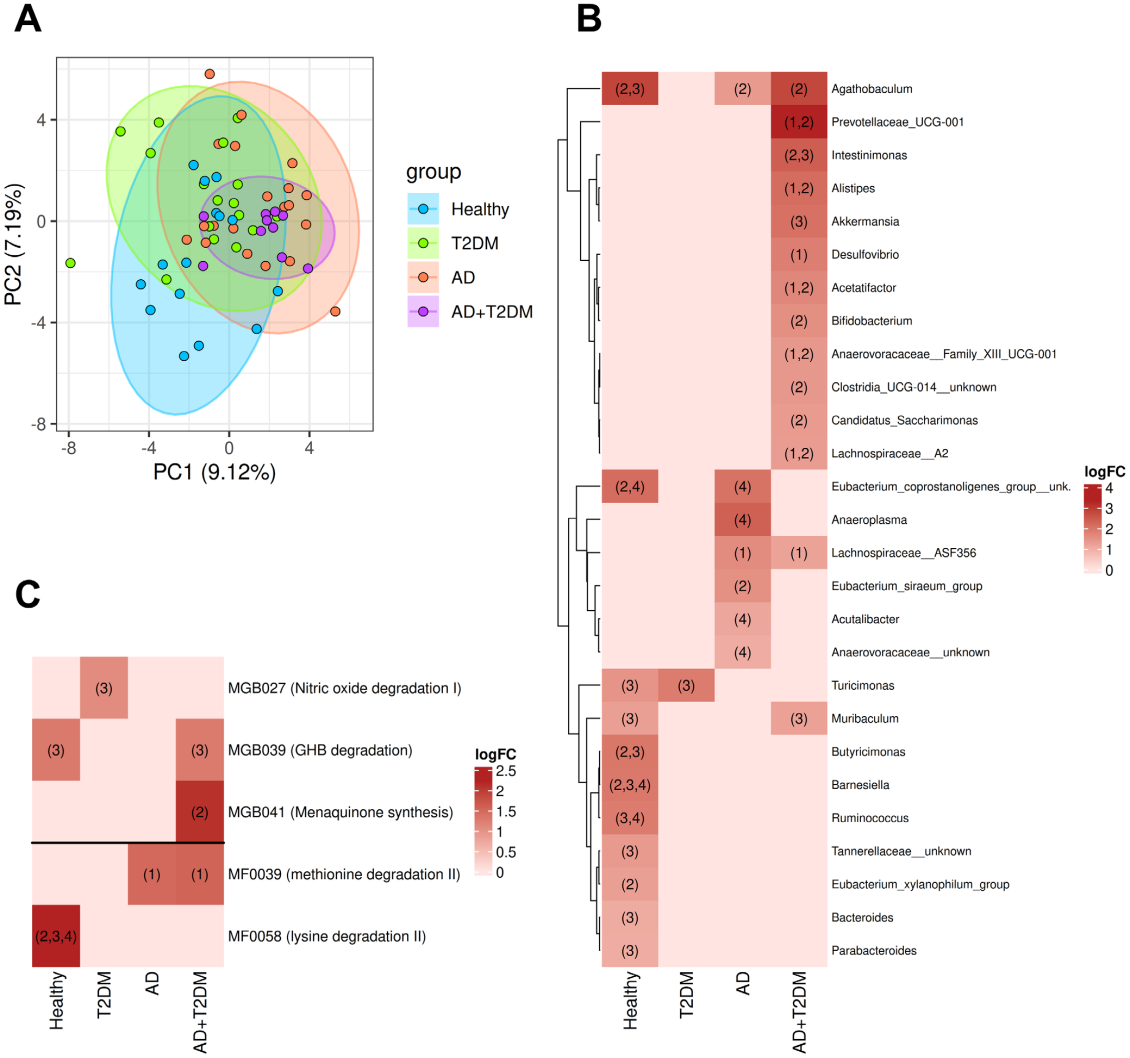
(A) Principal component analysis plot of mice gut microbial community. Each point represents an individual sample, colored according to the study group (Healthy, T2DM, AD, or AD+T2DM). Ellipses represent 95% confidence intervals, highlighting clustering patterns between disease and control groups. Axes indicate the proportion of variance explained by PC1 and PC2. (B) Heatmap of differentially abundant taxa across FMT groups. (C) Heatmap of differentially abundant functional modules across FMT groups. Each cell displays the LogFC of a feature abundance, indicating the magnitude and direction of differential abundance between study groups. Only taxa with LogFC > ±1 and adjusted *p* value < 0.05 are shown. Numbers within cells denote the comparison(s) where the feature is significantly enriched in the indicated group: (1) versus Healthy, (2) versus T2DM, (3) versus AD, (4) versus AD+T2DM.

The PCA among FMT groups showed that the first two principal components (PC1 and PC2) explained respectively 9.1% and 7.2% of the variance in microbial community structure. PCA plot shows partial group separation, with AD+T2DM microbiota being the most divergent (Figure 3A).

Global PERMANOVA revealed significant compositional differences among mouse FMT groups (Table S9). The experimental group explained 10.5% of the variance, indicating that FMT treatment significantly altered gut microbial composition (adjusted *p* = 0.001). Donor age also contributed significantly, explaining 2.8% of the variation in microbiota composition (adjusted *p* = 0.006), reflecting the potential influence of elderly‐associated microbiota on aging‐related host phenotypes in recipient mice. Overall, 84.6% of the variation remained unexplained by the tested factors, highlighting additional sources of variability in gut microbiota among individual mice. Pairwise PERMANOVA comparisons revealed that gut microbiota composition differed significantly between disease FMT groups and healthy recipients, with the AD+T2DM group accounting for the greatest variation (*R* ^2^ = 0.111, adjusted *p* = 0.002). These findings indicate that comorbid status exerted the strongest influence on the gut microbial community established in recipient mice.

Differential abundance analysis across FMT groups revealed donor‐dependent taxonomic shifts (Figure 3B). The AD+T2DM group displayed the most distinct microbial signature, with enrichment of several genera including *Prevotellaceae* UCG‐001, *Alistipes*, *Acetifactor*, *Anaerovoraceae* Family XIII UCG‐001, and *Lachnospiraceae* A2 (vs. healthy and T2DM), *Intestinimonas* (vs. T2DM and AD), *Akkermansia* and *Muribaculum* (vs. AD), *Desulfovibrio* (vs. healthy), *Bifidobacterium*, unknown *Clostridia* UCG‐014, *Agathobaculum*, and *Candidatus saccharimonas* (vs. T2DM). In contrast, healthy donor recipients showed enrichment in *Eubacterium coprostanoligenes* group (vs. T2DM and AD+T2DM), *Turicimonas*, *Muribaculum*, *Tannerellaceae* unknown genera, *Bacteroides*, and *Parabacteroides* (vs. AD), *Butyricimonas* and *Agathobaculum* (vs. T2DM and AD), *Barnesiella* (vs. all disease groups), *Ruminococcus* (vs. AD and AD+T2DM), *Eubacterium xylanophilum* (vs. T2DM). Other comparisons showed AD enrichment of *Eubacterium coprostanoligenes* group, *Anaeroplasma*, *Acutalibacter*, and *Anaerovoraceae* unknown genera (vs. AD+T2DM), and *Agathobaculum* (vs. T2DM).

Differential abundance analysis of functional modules revealed donor‐dependent metabolic differences among FMT groups (Figure 3C). Healthy donor recipients showed enrichment in γ‐hydroxybutyric acid degradation GBM (vs. AD) and lysine degradation II GMM (vs. other disease groups). In contrast, comorbid recipients showed enrichment in γ‐hydroxybutyric acid degradation, methionine degradation I GBM (vs. T2DM), and methionine degradation II GMM (vs. healthy). Furthermore, with nitric oxide degradation, GBM was enriched in T2DM versus AD, whereas methionine degradation II GMM was enriched in AD versus healthy.

Omnibus tests showed significant group differences in the expression of all tested neurotrophic and synaptic plasticity–related genes, WBC and GRAN blood counts, and the sniffing test discrimination index (Table S6). Detailed pairwise comparisons are provided in Table S10. Expression of neurotrophic and synaptic plasticity–related genes differed across groups, with AD and T2DM recipients consistently exhibiting higher levels compared with healthy and AD+T2DM groups. Hematological profiles also varied significantly, with AD and healthy mice showing higher WBC and GRAN counts than AD+T2DM and T2DM groups. Behaviorally, AD recipients displayed lower sniffing test discrimination index scores compared with the other groups.

### 3.4 Correlation Analyses

**FIGURE 4:**
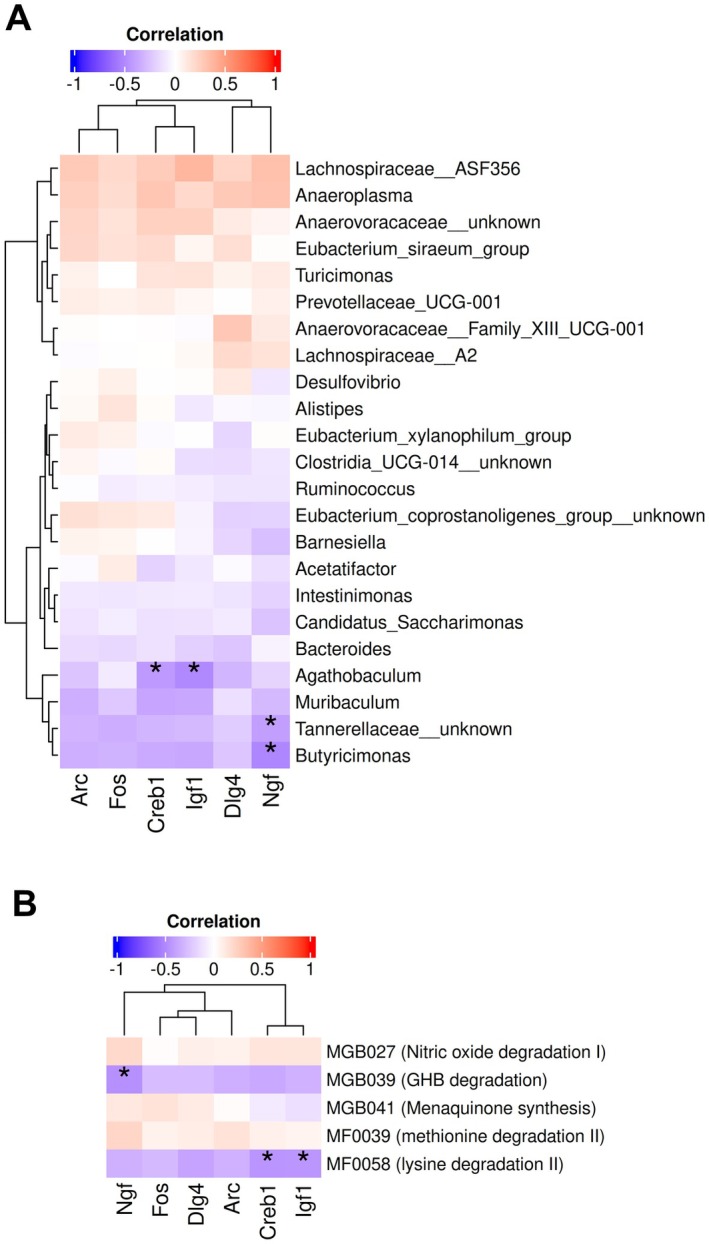
(A) Heatmap showing Spearman correlations between differentially abundant taxa across FMT groups and host hippocampal gene expression markers that also showed significant group‐level differences. (B) Heatmap showing Spearman correlations between differentially abundant functional modules across FMT groups and host hippocampal gene expression markers that also showed significant group‐level differences. Statistical significance was adjusted for multiple testing using the false discovery rate (FDR) method. Correlations exceeding |r| > 0.4 with FDR‐adjusted *p* values < 0.05 (indicated with * symbol) were considered significant.

Spearman correlation analysis was performed to assess associations between differentially abundant taxa or functional modules across FMT groups and host hippocampal gene expression markers or behavioral tests that showed significant group‐level differences. A significant negative correlation was observed between *Butyricimonas* and *Tannerellaceae* unknown genera with *Ngf* expression (Correlation = −0.525, adjusted *p* = 0.002; Correlation = −0.414, adjusted *p* = 0.038, respectively). Similarly, *Agathobaculum* was negatively correlated with both *Igf1* and *Creb1* expression (Correlation = −0.510, adjusted *p* = 0.002; Correlation = −0.438, adjusted *p* = 0.022) (Figure 4A, Table S11). No significant correlations were observed between behavioral tests that showed significant group‐level differences and differential abundant taxa (Table S12). For functional modules, γ‐hydroxybutyric acid degradation GBM was significantly negatively correlated with *Ngf* (Correlation = −0.481, adjusted *p* = 0.003). In addition, lysine degradation II GMM was negatively correlated with *Creb1* and *Igf1* (Correlation = −0.457, adjusted *p* = 0.003; Correlation = −0.454, adjusted *p* = 0.003) (Figure 4B, Table S13). No significant correlations were found between differentially abundant functional modules and behavioral tests that also showed significant group‐level differences (Table S14).

### 3.5 MetNet Reaction‐ and Substrate‐Level Findings in Mice (glmmTMB)

Using glmmTMB (logit link) at reaction (rxn) and substrate (subs) levels, we observed contrast‐specific depletion of secondary‐metabolite pathways in recipient mice (Figure S1). In AD+T2DM versus control, the largest negative logFC magnitudes clustered in flavonoid/isoflavonoid metabolism and exchange/demand reactions, whereas AD versus control and T2DM versus control showed smaller, subsystem‐specific shifts (primarily flavonoid, lignan, and phenol). Across contrasts, most top hits were negative (down‐regulated). Detailed reaction‐ and substrate‐level coefficients are provided in Table S15.

## 4 Discussion

A critical knowledge gap exists regarding the effects of FMT from donors with comorbid AD and T2DM, as no clinical or preclinical studies have directly evaluated its impact on neurometabolic outcomes in elderly populations. In this study, we investigated how age‐associated gut microbiota from elderly donors with AD, T2DM, and their comorbidity modulate host neurobiology, metabolic regulation, and behavior in FMT recipient mice. We observed that microbiota from comorbid donors induce the most severe dysbiosis, strongest suppression of hippocampal neurotrophic signaling, and greatest depletion of neuroprotective secondary metabolite pathways. These findings suggest that microbiota from elderly donors with AD, T2DM, and their comorbidity are associated with neurobiological alterations in recipient mice through convergent taxonomic, functional, and metabolic changes.

The analysis of human donor microbiota showed disease‐specific signatures. Although PCA analysis showed partial overlap among groups and substantial intra‐group variability, the global PERMANOVA confirmed that group membership explained a statistically significant portion of variance, while neither donor age nor nationality contributed significantly. This high inter‐individual variability is characteristic of human microbiome studies and reflects the inherent heterogeneity of gut microbial communities (Vujkovic‐Cvijin et al. 2020). Despite this variability, the disease‐specific signatures were sufficiently robust to produce distinct and statistically significant compositional differences, as observed in pairwise PERMANOVA results, where the AD+T2DM group showed the most pronounced dysbiosis, with significant compositional differences from healthy donors. Additionally, we observed an enrichment of taxa previously linked with metabolic stress and inflammation, including *Lachnoclostridium* and *Enterocloster* (Li and Li 2023; Mbaye et al. 2023). In contrast, we observed a reduction in butyrate‐producing taxa such as *Eubacterium siraeum* group and *Barnesiella*, which have been associated with epithelial barrier integrity and anti‐inflammatory activity (Lai et al. 2024; Liu et al. 2025). These taxonomic differences between healthy and disease‐derived donor microbiota suggest that comorbid disease in elderly donors may produce a synergistic disruption of gut–brain–metabolic homeostasis that exceeds the effects of either condition alone.

Functional profiling of human donor microbiota revealed modules involved in dopaminergic turnover, glutamate and cysteine metabolism, and carbohydrate fermentation altered in disease‐derived microbiota. These alterations suggest dysregulated microbial contributions to neurotransmitter balance, redox homeostasis, and inflammatory pathways (Gruenbaum et al. 2024; Hamamah et al. 2022; Y.‐T. Kim et al. 2024; Rowland et al. 2018). Together, these taxonomic and functional signatures indicate aging‐ and disease‐associated metabolic shift toward excitatory neurotransmission, sulfur–amino acid utilization, and reduced SCFA formation, processes potentially linked with both metabolic and neuroinflammatory stress.

The study demonstrates successful engraftment of the transplanted gut microbiota, with convergent evidence from community‐level metrics, indicator taxa, and functional profiling. Alpha diversity indices Shannon and Simpson were significantly higher in AD and AD+T2DM recipients compared with antibiotic‐treated controls, indicating restoration of microbial diversity following transplantation. Rather than reflecting a healthier community, this increased disease‐related diversity may represent colonization by metabolically flexible and stress‐tolerant taxa filling niches left by depleted beneficial commensals (Lozupone et al. 2012; Veseli et al. 2025). The distinct clustering of FMT samples away from controls and global PERMANOVA confirmed significant compositional differences between groups, demonstrating that donor microbiota induced measurable shifts in recipient gut ecology. Pairwise comparisons further showed that each donor group produced distinct microbial communities, with the AD+T2DM group exhibiting the strongest compositional shift, indicating that donor disease status and age‐associated microbial profiles were successfully transmitted to recipients.

The enrichment of specific taxa in FMT recipients, including *Lachnoclostridium*, linked to inflammatory signaling and altered carbohydrate metabolism (Li and Li 2023), and *Oscillibacter*, associated with anxiety‐like behavior (Chi et al. 2024), alongside with depletion of genera such as *Roseburia*, and *Eubacterium* spp., SCFA producers and regulators of gut–brain homeostasis (Nie et al. 2021; Rowland et al. 2018), suggest that FMT induced marked and aging‐ and disease‐specific alterations in the gut microbiota of recipient mice, confirming that donor microbial composition drives functionally relevant remodeling of host gut ecology (He et al. 2022; Schmidt et al. 2022). Disease‐derived FMTs promoted upregulation of pathways involved in dopaminergic and acetate metabolism, lactate utilization, urea degradation, and menaquinone synthesis, suggesting a shift toward energy‐intensive fermentation and redox‐active metabolism. In contrast, control mice showed higher representation of GABA synthesis, glutamate degradation, and fatty acid β‐oxidation modules, indicating a more metabolically stable, inhibitory‐neurotransmitter‐supporting microbial network.

Engraftment extended beyond taxonomic colonization to influence host neurobiology, immunity, and gastrointestinal physiology. The higher *Creb1* expression in controls' hippocampus compared with healthy and comorbid recipients suggests that microbiota reconstitution, whether from healthy or disease‐derived donors, possibly modulates hippocampal transcriptional programs through the microbiota‐gut–brain axis (Tang et al. 2021). Furthermore, higher LYM and WBC blood counts in controls compared with selected FMT recipients likely reflect the interplay between antibiotic‐induced perturbation and subsequent microbiota‐driven reconstitution (Ekmekciu et al. 2017a, 2017b; Josefsdottir et al. 2017). Finally, the higher cecum weight observed in FMT recipients compared with controls can be considered a physiological marker of successful microbiota reconstitution after antibiotic‐induced depletion. The restoration of cecum size and function after FMT thus indicates that the transplanted microbiota has re‐established metabolic activity and ecological niches disrupted by antibiotics (Ji et al. 2017; Suez et al. 2018). These convergent taxonomic, functional, and physiological signatures collectively demonstrate that donor microbiota successfully colonized recipient mice and exerted measurable effects on host biology.

Beyond demonstrating successful engraftment, comparative microbiota profiling across FMT groups revealed that donor disease status fundamentally shaped recipient microbial communities and downstream host phenotypes. The significant beta diversity differences indicate that the compositional structure of each recipient's microbiota was shaped by donor disease status. The AD+T2DM versus healthy comparison showed the greatest divergence, demonstrating that comorbid disease‐associated microbiota produces the most distinct recipient community. This supports the concept that FMT clinical success is related to the degree of donor microbial engraftment and that donor microbiota composition fundamentally determines recipient outcomes (Ianiro et al. 2022; Porcari et al. 2023). The significant contribution of donor age to recipient microbiota variation suggests that elderly‐associated microbial signatures are transmissible and may drive aging‐related phenotypes in recipients (Khan et al. 2024). The transmissibility of these aging‐associated signatures suggests that the gut microbiota may serve as a modifiable factor in age‐related neurodegeneration, with important implications for therapeutic interventions targeting the microbiota‐gut–brain axis in elderly populations (Chidambaram et al. 2022).

Comparative microbiota profiling across FMT groups showed the enrichment of pro‐inflammatory and metabolically active taxa in AD+T2DM recipients, including *Prevotellaceae* UCG‐001, *Alistipes*, and *Akkermansia*, reflecting the transmission of disease‐associated dysbiosis. These taxa have been linked to LPS production, bile acid and aromatic compound dysmetabolism, and mucin degradation (Derrien et al. 2004; Larsen 2017; Parker et al. 2020). In contrast, healthy donor recipients showed enrichment of taxa including *Butyricimonas*, *Ruminococcus*, and *Eubacterium coprostanoligenes* group, associated with SCFA production, anti‐inflammatory activity, and maintenance of gut barrier integrity (H.‐C. Chen et al. 2023; Deleu et al. 2021; Igudesman et al. 2022). This comparison between healthy and disease‐derived recipient profiles suggest that microbiota from healthy elderly individuals may confer a protective taxonomic signature, while disease‐derived microbiota may transmit a pro‐inflammatory, metabolically dysregulated community.

Functional profiling indicated enrichment of methionine degradation pathways in AD+T2DM recipients, suggesting enhanced microbial sulfur–amino acid turnover and increased homocysteine and oxidative stress metabolite production, processes previously associated with accelerated aging, neurodegeneration, and vascular dysfunction (Smith et al. 2018; Wu et al. 2022). Enrichment of γ‐hydroxybutyric acid and nitric oxide degradation modules in disease FMTs may reflect reduced microbial support for inhibitory neurotransmission and endothelial signaling (Cryan et al. 2019; Grishin et al. 2016). In contrast, healthy FMTs maintained lysine degradation and urea recycling pathways, linked to butyrate and polyamine synthesis essential for nitrogen balance and mucosal integrity maintenance (Bui et al. 2015; Leggett et al. 2022; Moinard et al. 2005; Pugin et al. 2017). The preservation of these neuroprotective metabolic pathways in healthy recipients highlights the functional consequences of donor health status on recipient metabolic capacity.

The hippocampal neurotrophic gene expression reflects microbiota‐driven effects. Mice colonized with AD and AD+T2DM microbiota showed marked reductions in *Ngf*, *Igf1*, *Arc*, *Fos*, and *Creb1* expression, indicating impaired neurotrophic signaling and reduced synaptic activity (Benito and Barco 2010; Lonze and Ginty 2002). The strongest suppression occurred in the AD+T2DM recipients, particularly for *Creb1* and *Arc*, suggesting that comorbid microbiota may impair neuronal plasticity signaling. The T2DM group retained relatively higher expression of these genes, possibly reflecting compensatory insulin‐related trophic signaling in the hippocampus (Kellar and Craft 2020).

Behavioral assessments showed reduced olfactory discrimination and an anhedonia‐like reduction in motivational drive, along with increased daytime locomotor activity in mice receiving AD microbiota, phenotypes often associated with impaired attention, disrupted circadian regulation, and hippocampal dysfunction (Musiek and Holtzman 2016; Wesson et al. 2010). The reduced olfactory discrimination in AD recipients is particularly relevant, as olfactory dysfunction is among the earliest clinical manifestations of Alzheimer's disease (Yan et al. 2022), and gut microbiota composition has been shown to influence olfactory epithelium physiology and olfactory preferences. These behavioral findings provide functional evidence that disease‐derived microbiota can induce phenotypes relevant to early AD pathophysiology (Naudon et al. 2020).

Correlation analyses provided mechanistic insights into the relationships between donor‐dependent microbiota features and host neurobiological outcomes. The negative correlation between *Butyricimonas*, *Tannerellaceae* spp., and *Agathobaculum* abundance, with *Ngf*, *Igf1*, and *Creb1* hippocampal expression, suggests potential suppression of neurotrophic signaling through microbial metabolic or inflammatory pathways (Bercik et al. 2011; Y. Chen et al. 2021; Guzzetta et al. 2022). The negative correlation between γ‐hydroxybutyric acid degradation and lysine degradation functional modules with *Ngf*, *Creb1*, and *Igf1* hippocampal expression suggests altered excitatory–inhibitory neurotransmitter balance or redox modulation (Bui et al. 2015; Valles‐Colomer et al. 2019). These patterns support a mechanistic model in which age‐ and disease‐associated microbial catabolism of neuroactive metabolites reduces substrate availability for trophic and synaptic signaling in the hippocampus.

Metabolic‐network analysis revealed broad downregulation of secondary metabolite biosynthesis and exchange reactions in recipient mice, particularly AD+T2DM. Depletion in flavonoid, isoflavonoid, lignan, and phenolic pathways aligns with reports of reduced gut‐derived phenolics in metabolic disease and impaired biotin/phenolic metabolism in AD (Belda et al. 2022; Lohr et al. 2020; Pallister et al. 2017). Lignan supplementation has improved cognition in AD models, consistent with observed depletion of lignan‐related reactions (Jia et al. 2024). Thus, both microbial composition and metabolic capacity converge to reduce antioxidant and neuroprotective metabolite pools in aging‐ and disease‐associated microbiota recipients.

This study has several strengths, including the use of antibiotic‐treated mice to isolate microbiota‐specific effects, the parallel integration of human donor profiling with taxonomic, functional, and metabolic network analyses in recipients, and the combined assessment of molecular, immune, and behavioral outcomes. The inclusion of a comorbid AD+T2DM group, a clinically relevant but understudied phenotype, allowed us to test additive versus synergistic microbiota effects that are not captured in single‐disease models.

Nonetheless, limitations must be acknowledged. The use of female human donors and male mouse recipients introduces potential sex‐related confounds. Female donors were included because they were the only individuals meeting all eligibility criteria, and all were postmenopausal with stable hormonal status, minimizing short‐term reproductive endocrine variability on microbiota composition (Santos‐Marcos et al. 2018; da Silva et al. 2025; H. Zhao et al. 2019). Transferring microbiota from hormonally stable postmenopausal women into cycling female mice could have introduced substantial variability due to estrous‐cycle‐dependent hormonal changes in recipients, potentially confounding interpretation of microbiota‐driven effects. Male mice were therefore selected to reduce biological variation and improve reproducibility. Notably, recent clinical evidence indicates that donor‐recipient sex concordance does not significantly affect FMT outcomes (Karmisholt Grosen et al. 2025). Nevertheless, sex‐specific effects cannot be excluded, and future studies should include both male and female recipients to directly evaluate the impact of donor‐recipient sex matching on neurobiological and metabolic outcomes. Changes in hippocampal gene expression were assessed at the mRNA level without protein‐level validation or functional histological analyses. While mRNA expression provides insight into transcriptional regulation, post‐transcriptional modifications may result in discordance between transcript and protein levels. The absence of spatial transcriptomic data limits our ability to determine which specific cell populations within the hippocampus are most affected by microbiota‐driven changes. Colonization of antibiotic‐treated mice may not fully recapitulate long‐term host‐microbe co‐adaptation; sample size per donor group may limit generalizability; and functional profiles represent microbial potential rather than measured metabolite levels. The absence of targeted metabolomics or region‐specific neurophysiology currently restricts mechanistic resolution.

In summary, integrated taxonomic, functional, and metabolic analyses show that microbiota from disease donors, especially those with comorbid AD+T2DM, reshape host microbial communities toward reduced SCFA and GABA production, increased sulfur–amino‐acid and dopaminergic metabolism, and diminished secondary‐metabolic capacity, reflecting aging‐associated functional decline. These alterations coincide with impaired hippocampal neurotrophic signaling, behavioral dysfunction, and negative microbiota–gene correlations. Together, these findings support a mechanistic model in which age‐ and disease‐associated gut dysbiosis acts as a compounding factor linking metabolic dysfunction and neurodegeneration through convergent effects on microbial metabolism, neurotrophic pathways, and the microbiota‐gut–brain axis.

Future studies should pair FMT models with targeted metabolomics and brain‐region–specific transcriptomics to validate the causal metabolites and signaling pathways implicated here. Incorporating targeted metabolomics, protein quantification, immunohistochemistry, and region‐specific neurophysiological recordings may help validate whether transcriptional changes translate into meaningful physiological and behavioral effects. Longitudinal colonization studies in conventionally raised mice, along with gnotobiotic reconstruction using defined microbial consortia, will help determine which taxa or modules drive neurotrophic suppression. Integrating these approaches with clinical cohorts, particularly individuals with comorbid metabolic and neurodegenerative conditions, will be essential to translate microbiota‐based interventions into therapeutic strategies for aging‐related cognitive and metabolic decline.

## Author Contributions

D.B. conceived and A.B. designed the study. Patient enrollment, clinical characterization, sample collection, and sample processing were performed by R.L., J.D., K.Ł., R.R., D.K., G.N., T.P., K.S., K.K., J.K. Animal experiments were conducted by J.M., K.K. and E.K. A.A. processed sequencing data and performed taxonomic and functional microbiota profiling and statistical analyses. G.A. performed microbiota metabolic profiling inference. A.A. drafted the manuscript with critical revisions from A.B., D.B. and J.M.‐P. All authors approved the final version to be submitted for publication.

## Funding

This work was supported by a grant from the Research Council of Lithuania (LMTLT), Agreement No. S‐PD‐24‐95.

## Conflicts of Interest

The authors declare no conflicts of interest.

## Supporting information


**Figure S1.1:** Visual representation of the open field maze. The red line outlines the central zone; note that this line is not visible during the actual test.
**Figure S1.2:** Visual representation of plus maze.
**Figure S1.3:** Visual representations of the Novel Object Recognition and Short‐Term Memory test maze. (A) Stage 1: no objects are present in the maze (habituation); (B) Stage 2: two identical objects are placed at the ends of the maze arms (familiarization); (C) Stage 3: one object remains the same as in Stage 2, while the other is replaced with a novel, previously unseen object (test phase).
**Figure S1.5:** Visual representation of the Y‐maze. Letters indicate the labeled arms.
**Figure S1.6:** Visual example of the Running Wheel test.
**Figure S1.7:** Visual example of the social interaction test. (A) Stage 1: no objects are placed behind the barriers; (B) Stage 2: an object remains in one compartment, and a mouse is placed in the other; (C) Stage 3: the mouse introduced in Stage 2 remains, while the object is replaced by a novel mouse previously unfamiliar to the test mouse.
**Table S1:** General characteristics of human donors.
**Table S2:** Pairwise differences in alpha diversity indices Chao1, Shannon, and Simpson across human FMT donors groups. Kruskal–Wallis tests followed by Dunn's post hoc comparisons (Benjamini–Hochberg adjusted) was used to identify significant differences. Significant adjusted *p* < 0.05.
**Table S3:** PERMANOVA results obtained by testing the effects of group, age, and nationality on human gut microbiota composition (Aitchison distances). *p* values were adjusted using the Benjamini–Hochberg procedure. Significant adjusted *p* < 0.05.
**Table S4:** Pairwise differences in alpha diversity indices Chao1, Shannon, and Simpson between control versus FMT recipients. Kruskal–Wallis tests followed by Dunn's post hoc comparisons (Benjamini–Hochberg adjusted) was used to identify significant differences. Significant adjusted *p* < 0.05 in bold.
**Table S5:** PERMANOVA results obtained by testing differences in gut microbiota composition (Aitchison distances) among mice experimental groups. *p* values were adjusted using the Benjamini–Hochberg procedure. Significant *p* < 0.05.
**Table S6:** Group differences in hippocampal gene expression markers, blood count, and behavioral measures among experimental mice experimental groups.
**Table S7:** Pairwise comparison in hippocampal gene expression markers, blood count, and behavioral measures between mice control group versus FMT recipients.
**Table S8:** Pairwise differences in alpha diversity indices Chao1, Shannon, and Simpson across FMT recipients. Kruskal‐Wallis tests followed by Dunn's post hoc comparisons (Benjamini–Hochberg adjusted) was used to identify significant differences. Significant adjusted *p* < 0.05.
**Table S9:** PERMANOVA results obtained by testing the effects of group, age, and nationality on FMT donors gut microbiota composition (Aitchison distances). *p* values were adjusted using the Benjamini–Hochberg procedure. Significant adjusted *p* < 0.05.
**Table S10:** Pairwise comparison in hippocampal gene expression markers, blood count, and behavioral measures across mice FMT groups.
**Table S11:** Results of Spearman correlations between differentially abundant taxa across FMT groups and host hippocampal gene expression markers that also showed significant group‐level differences. Statistical significance was adjusted for multiple testing using the false discovery rate (FDR) method. Correlations exceeding |*r*| > 0.4 with FDR‐adjusted *p* values < 0.05 were considered significant (in bold).
**Table S12:** Results Spearman correlations between differentially abundant taxa across FMT groups and behavioral tests scores that also showed significant group‐level differences. Statistical significance was adjusted for multiple testing using the false discovery rate (FDR) method. Correlations exceeding |*r*| > 0.4 with FDR‐adjusted *p* values < 0.05 were considered significant.
**Table S13:** Results Spearman correlations between differentially abundant functional modules across FMT groups and host hippocampal gene expression markers that also showed significant group‐level differences. Statistical significance was adjusted for multiple testing using the false discovery rate (FDR) method. Correlations exceeding |*r*| > 0.4 with FDR‐adjusted *p* values < 0.05 were considered significant (in bold).
**Table S14:** Results Spearman correlations between differentially abundant functional modules across FMT groups and behavioral tests scores that also showed significant group‐level differences. Statistical significance was adjusted for multiple testing using the false discovery rate (FDR) method. Correlations exceeding |*r*| > 0.4 with FDR‐adjusted *p* values < 0.05 were considered significant.
**Figure S1:** (A) Top glmmTMB_logit features by contrast (mice). Dot plot of metabolite/substrate‐level features colored by metabolic subsystem; point size encodes |logFC|. Contrasts: T2DM versus control, and AD+T2DM versus control. (B) Top glmmTMB_logit reaction‐level features by contrast (mice), faceted by biochemical subsystem. Point size encodes |logFC| and color denotes signed logFC. Reactions with the largest magnitudes are labeled in‐panel. Contrasts: T2DM versus control, and AD+T2DM versus control.
**Table S15:** Top glmmTMB_logit features per contrast (mice) with annotations. [GA] Columns include contrast, feature identifier, subsystem, log fold change (logFC), adjusted *p* value (BH), and any available annotations.

## Data Availability

Data that support the findings of this study are available from the corresponding authors upon request.
