# Supplementary material for: Comorbid Alzheimer's Disease and Type 2 Diabetes Microbiota Shape Age‐Associated Gut–Brain Axis Profiles
Source: Aging Cell. 2026 Apr 21;25(5):e70488. doi: 10.1111/acel.70488 (PMC13099590; doi:10.1111/acel.70488)
Supplement: Supplementary file 1 — Figure S1.1: Visual representation of the open field maze. The red line outlines the central zone; note that this line is not visible during the actual test. Figure S1.2: Visual representation of plus maze. Figure S1.3: Visual representations of the Novel Object Recognition and Short‐Term Memory test maze. (A) Stage 1: no objects are present in the maze (habituation); (B) Stage 2: two identical objects are placed at the ends of the maze arms (familiarization); (C) Stage 3: one object remains the same as in Stage 2, while the other is replaced with a novel, previously unseen object (test phase). Figure S1.5: Visual representation of the Y‐maze. Letters indicate the labeled arms. Figure S1.6: Visual example of the Running Wheel test. Figure S1.7: Visual example of the social interaction test. (A) Stage 1: no objects are placed behind the barriers; (B) Stage 2: an object remains in one compartment, and a mouse is placed in the other; (C) Stage 3: the mouse introduced in Stage 2 remains, while the object is replaced by a novel mouse previously unfamiliar to the test mouse. Table S1: General characteristics of human donors. Table S2: Pairwise differences in alpha diversity indices Chao1, Shannon, and Simpson across human FMT donors groups. Kruskal–Wallis tests followed by Dunn's post hoc comparisons (Benjamini–Hochberg adjusted) was used to identify significant differences. Significant adjusted p < 0.05. Table S3: PERMANOVA results obtained by testing the effects of group, age, and nationality on human gut microbiota composition (Aitchison distances). p values were adjusted using the Benjamini–Hochberg procedure. Significant adjusted p < 0.05. Table S4: Pairwise differences in alpha diversity indices Chao1, Shannon, and Simpson between control versus FMT recipients. Kruskal–Wallis tests followed by Dunn's post hoc comparisons (Benjamini–Hochberg adjusted) was used to identify significant differences. Significant adjusted p < 0.05 in bold. Table S5: PERMANOVA results obta [file ACEL-25-e70488-s001.docx]

**Comorbid Alzheimer’s disease and type 2 diabetes microbiota shape age-associated gut–brain axis profiles**

**SUPPLEMENTAL MATERIAL**

**Supplementary Methods**

**1 Behavioral tests**

*1.1 Open Field Test*

This test is designed to assess anxiety, exploratory behavior, and locomotor activity in rodents. It is based on the natural aversion of rodents to brightly lit, open environments.

Mice were placed in a bright (500 lx) open field (40 cm x 40 cm) box that was unfamiliar to them for 10 min.

Mice were placed in a brightly lit (500 lx), open-field arena measuring 40 cm × 40 cm, which was unfamiliar to them. Each trial lasted 10 minutes. The mice were introduced at the center of the field, and their behavior was recorded for subsequent analysis.

Specific parameters were analyzed, including the duration, velocity, and frequency of visits in the central zone (defined as 1/4 of the total area) and the peripheral zone (the remaining area) (**Figure S2.1**). A camera mounted above the arena recorded all activity, and behavioral data were analyzed using Olympus Viewer 3 software (OM Digital Solutions Corporation, Japan). The software tracked time spent in each zone, overall activity, and the total distance traveled.

Rodents exhibiting higher levels of anxiety tend to explore less and remain in perceived safe areas, such as the periphery. Since the center of the maze is more exposed and perceived as less safe, highly anxious mice typically avoid it and stay close to the walls.


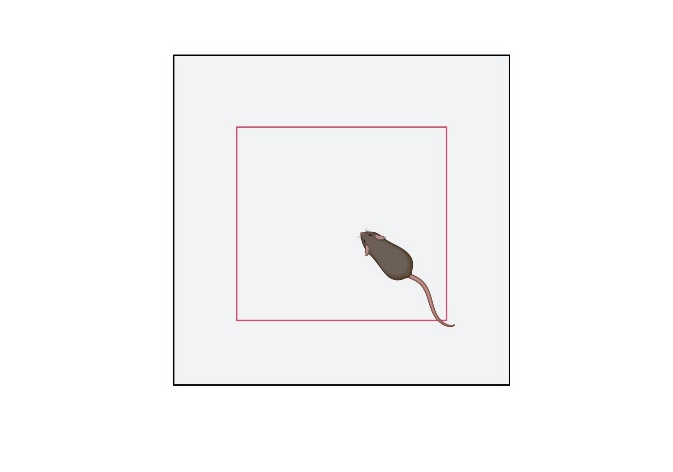


**Figure S1.1** Visual representation of the open field maze. The red line outlines the central zone; note that this line is not visible during the actual test.

*1.2 Elevated Plus Maze Test*

The elevated plus maze test is used to assess anxiety levels and exploratory behavior in rodents. The apparatus consists of two open arms and two closed arms (each measuring 25.5 cm × 6 cm) arranged in a plus configuration, with a central zone measuring 4.5 cm × 4.5 cm (**Figure S1.2**). The closed arms are enclosed by 10 cm high side walls. The entire maze is elevated 66 cm above the floor. Lighting is set to 50 lx in the open arms and 20 lx in the closed arms.

At the start of the test, the mouse is placed in the central zone facing an open arm and allowed to explore the maze freely for 5 minutes. After the trial, the mouse is returned to its home cage. A video camera positioned above the maze records all activity.

During video analysis, the following parameters are recorded: the number of entries into open arms, time spent in open and closed arms, entries into the distal third of the open arms, and time spent in these extremities.

The test is based on the natural aversion of mice to open and elevated spaces. While both open and closed environments evoke exploratory drive, avoidance of the open arms is interpreted as a behavioral indicator of anxiety (Komada, Takao, & Miyakawa, 2008).


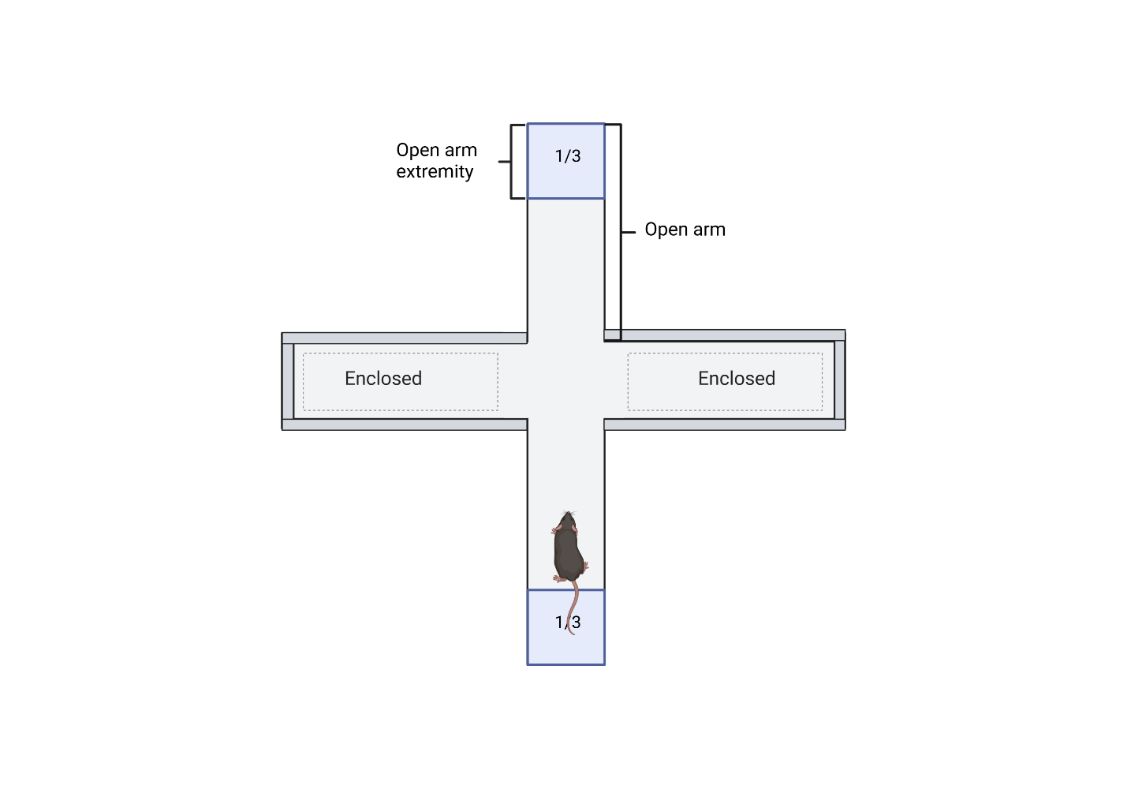


**Figure S1.2** Visual representation of plus maze.

*1.3 Novel Object Recognition Test*

The Novel Object Recognition (NOR) test assesses a mouse’s ability to recognize and remember new objects, leveraging its innate tendency to explore novel stimuli. The test is conducted in a V-shaped maze, with two connected arms (each 30 cm × 6 cm, walls 10 cm high). Test objects are placed at the distal ends of each arm. The arena is illuminated at 15 lx to create a low-stress, comfortable environment.

The test is conducted over three days and includes three stages:

Stage 1 (Day 1): Habituation. The mouse is placed at the center of the maze and allowed to explore the empty arena (**Figure S1.3 A**) for 9 minutes. It is then returned to its home cage for 24 hours.

Stage 2 (Day 2): Familiarization. The mouse is again placed at the center of the maze, now containing two identical objects positioned at the ends of each arm **(Figure S1.3 B**). It is allowed to explore for 9 minutes before being returned to its cage for another 24 hours.

Stage 3 (Day 3): Test. One of the familiar objects is replaced with a novel object (**Figure S1.3 C**). The mouse is placed at the center of the maze and allowed to explore both the familiar and novel objects for 9 minutes. Mouse behavior is recorded by a video camera, and the researcher quantifies the time spent exploring each object.

To assess recognition memory, a Discrimination Index (DI) is calculated based on exploration times using the following formula:

$$\text{DI}=\frac{\left( T_{N}-T_{O} \right)}{\left( T_{N}+T_{O} \right)}$$

In this formula, T_N_ represents time spent analyzing the new object, and T_O_ represents time spent exploring the familiar (old) object. Higher DI values indicate better recognition memory. A preference for the novel object suggests effective recognition and discrimination abilities. If the mouse remembers the previously encountered object, it will spend less time investigating it during the test phase. Conversely, impaired memory is indicated when the mouse shows equal or greater interest in the familiar object.


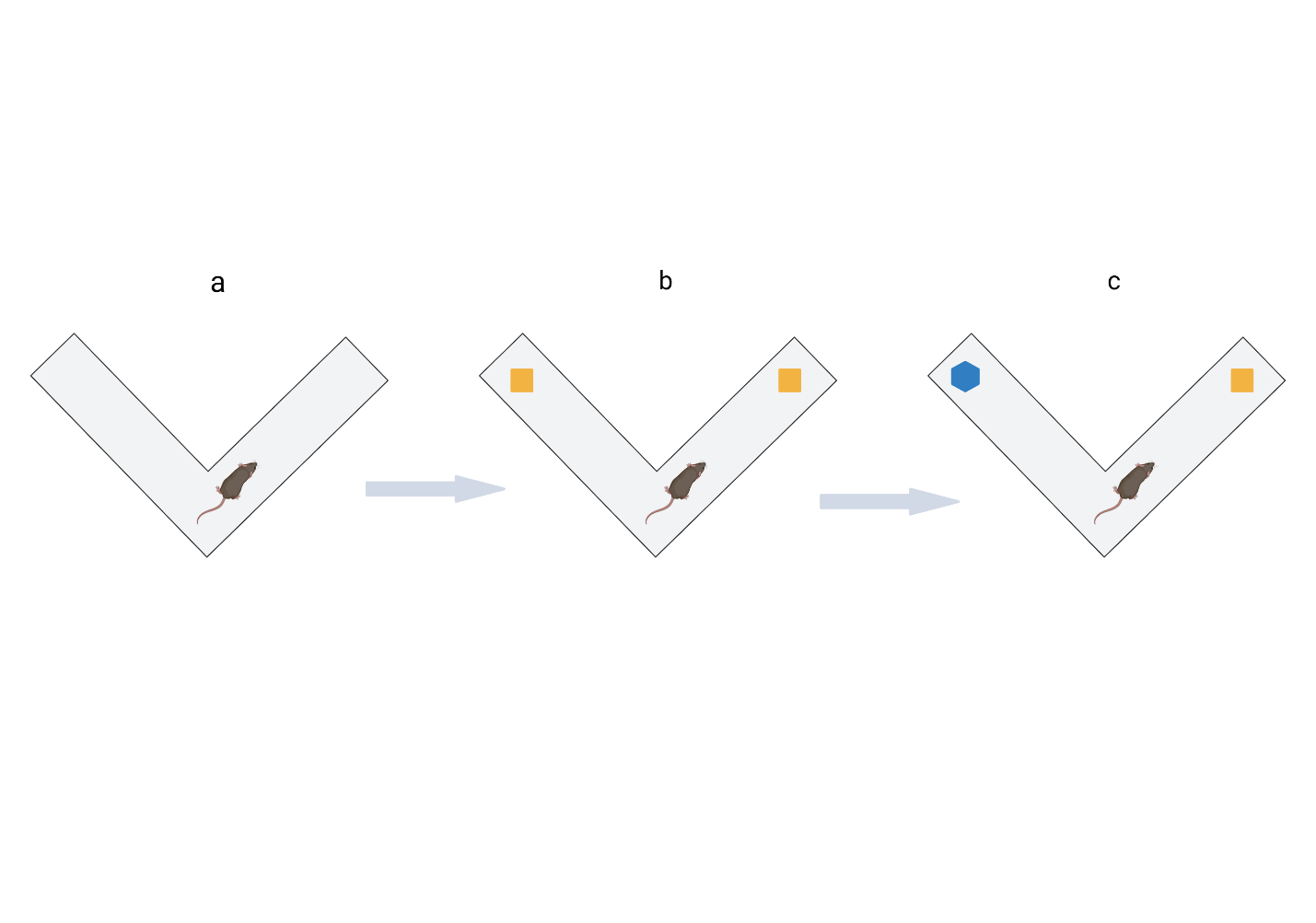


**Figure S1.3** Visual representations of the Novel Object Recognition and Short-Term Memory test maze. (A) Stage 1: no objects are present in the maze (habituation); (B) Stage 2: two identical objects are placed at the ends of the maze arms (familiarization); (C) Stage 3: one object remains the same as in Stage 2, while the other is replaced with a novel, previously unseen object (test phase).

*1.4 Short-Term Memory Test*

The short-term memory test is conducted under the same conditions as the Novel Object Recognition (NOR) test (see Section 2.3). The measured parameters and calculation methods are identical. The only difference is the interval between Stage 2 and Stage 3, which is reduced to 3 hours instead of 24 hours.

*1.5 Y-Maze Test*

The Y-maze test is used to assess working memory and exploratory activity in mice. The apparatus consists of a Y-shaped maze with three connected arms (each 40 cm long, 9 cm wide, with 10 cm high walls) and a central intersection zone. The maze is illuminated at 15 lx to ensure a low-stress, comfortable environment (**Figure S1.5**).

During the test, the mouse is placed at the end of one arm and allowed to explore freely for 9 minutes. A video camera positioned above the maze records its behavior. After the session, the footage is analyzed to record the sequence of arm entries.

Exploratory activity is assessed by counting arm entries, while working memory is evaluated by calculating the percentage of spontaneous alternations, defined as consecutive entries into three different arms without repetition.

Working memory performance is expressed as the Spontaneous Alternation Percentage (SAP) using the following formula:

$$SAP\%=\frac{NoA}{TPA-2}*100$$

Where NoA represents the number of observed alternations, and TPA refers to the total possible alternations. Alternations are determined based on sequential arm entries: for example, if a mouse enters arms in the sequence A → B → C, it is counted as one alternation. A sequence such as B → C → A → B → C would yield three alternations. In contrast, a repetitive sequence like B → C → B → C → B results in zero alternations, as no unique three-arm sequences occur.

This test leverages the rodent’s natural exploratory tendency to investigate novel environments rather than revisiting previously explored areas. Mice with intact working memory typically remember which arms they have already visited and preferentially explore new ones (Kraeuter, Guest, & Sarnyai, 2019).


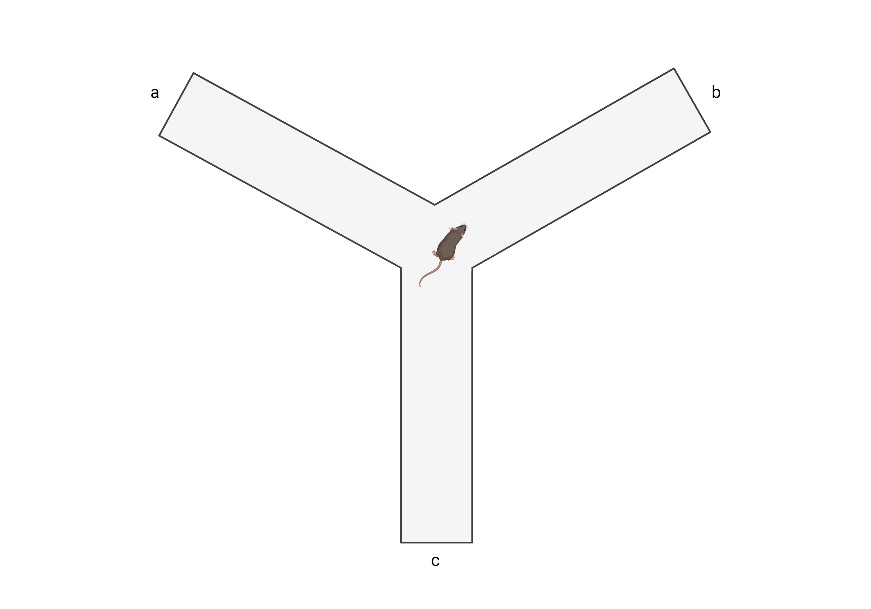


**Figure S1.5** Visual representation of the Y-maze. Letters indicate the labeled arms.

*1.6 Running Wheel Activity Test*

Voluntary running on a rotating wheel is widely used to study physical activity and behavior in mice. On the test day, the mouse is placed in a cage (20 cm × 26 cm) equipped with a rotating wheel (12 cm diameter) (**Figure S1.6**). Food and water are provided ad libitum.

During the first hour, the mouse is allowed to acclimate to the new environment, and running activity is not recorded. After this acclimation period, the LabChart software (ADInstruments, New Zealand) is activated to record the number and precise timing of wheel rotations. The mouse remains in the cage with access to the running wheel for a full 24-hour cycle, consisting of 12 hours of light and 12 hours of darkness.

At the end of the test, the researcher uses LabChart to calculate the total number of rotations performed during the light period, the dark period, and the combined 24-hour period. Laboratory mice typically exhibit spontaneous running behavior when provided with a wheel; however, their physical activity tolerance may decline due to chronic diseases or aging (Manzanares, Brito-da-Silva, & Gandra, 2019).


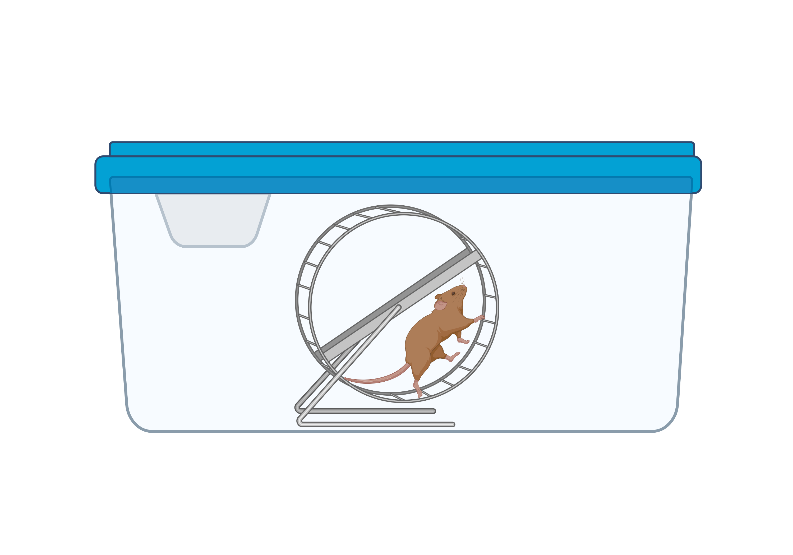


**Figure S1.6** Visual example of the Running Wheel test.

*1.7 Social Maze Test*

The Social Maze test evaluates two parameters in mice: sociability and preference for social novelty. The apparatus consists of a V-shaped maze with two connected arms (each measuring 30 cm × 6 cm, with 10 cm high walls). Each arm ends with a barrier that separates the test mouse from either an inanimate object (4 cm wide, 6 cm high; see figure) or a live conspecific (another mouse). The barrier’s grid spacing (10 mm) allows the test mouse to sniff and explore through the barrier, with or without direct contact. Illumination is set to 15 lx to ensure a comfortable, low-stress environment.

The test consists of three consecutive stages completed within 15 minutes:

Stage 1 (Habituation): The mouse is placed in the center of the maze and allowed to explore freely for 5 minutes, with no objects behind the barriers (**Figure S1.7 A**).

Stage 2 (Sociability Test): One arm contains an artificial object, and the other contains an unfamiliar live mouse (**Figure S1.7 B**). The test mouse explores for 5 minutes while the time spent investigating each stimulus is recorded.

Stage 3 (Social Novelty Recognition Test): The artificial object is replaced by a novel unfamiliar mouse, while the previously introduced mouse remains in place (**Figure S1.7 C**). The test mouse explores for 5 minutes, and exploration times for both the familiar and novel mouse are recorded.

Using the exploration times from Stages 2 and 3, discrimination indices for sociability and novelty preference are calculated:

$$\text{DI}= \frac{\text{t1}-\text{t2}}{\text{t1 + t2}}$$

When counting the sociability discrimination index, t_1_ represents time spent analyzing the unfamiliar mouse during the second stage of the test, and t_2_ represents the time spent inspecting the artificial object. For the novelty preference index, t_1_ is the time spent inspecting the new mouse, while t_2_ is the time spent with the familiar mouse.

The second stage assesses the mouse’s ability to distinguish between a living and a non-living stimulus, as well as its tendency to engage in social interaction. The third stage evaluates the mouse’s ability to recognize and remember familiar versus unfamiliar conspecifics, reflecting more complex memory and social recognition processes (Martínez-Torres, Gomis-González, Navarro-Romero, Maldonado, & Ozaita, 2019). A higher discrimination index indicates a stronger ability to differentiate between a mouse and an object or between a familiar and a novel mouse.


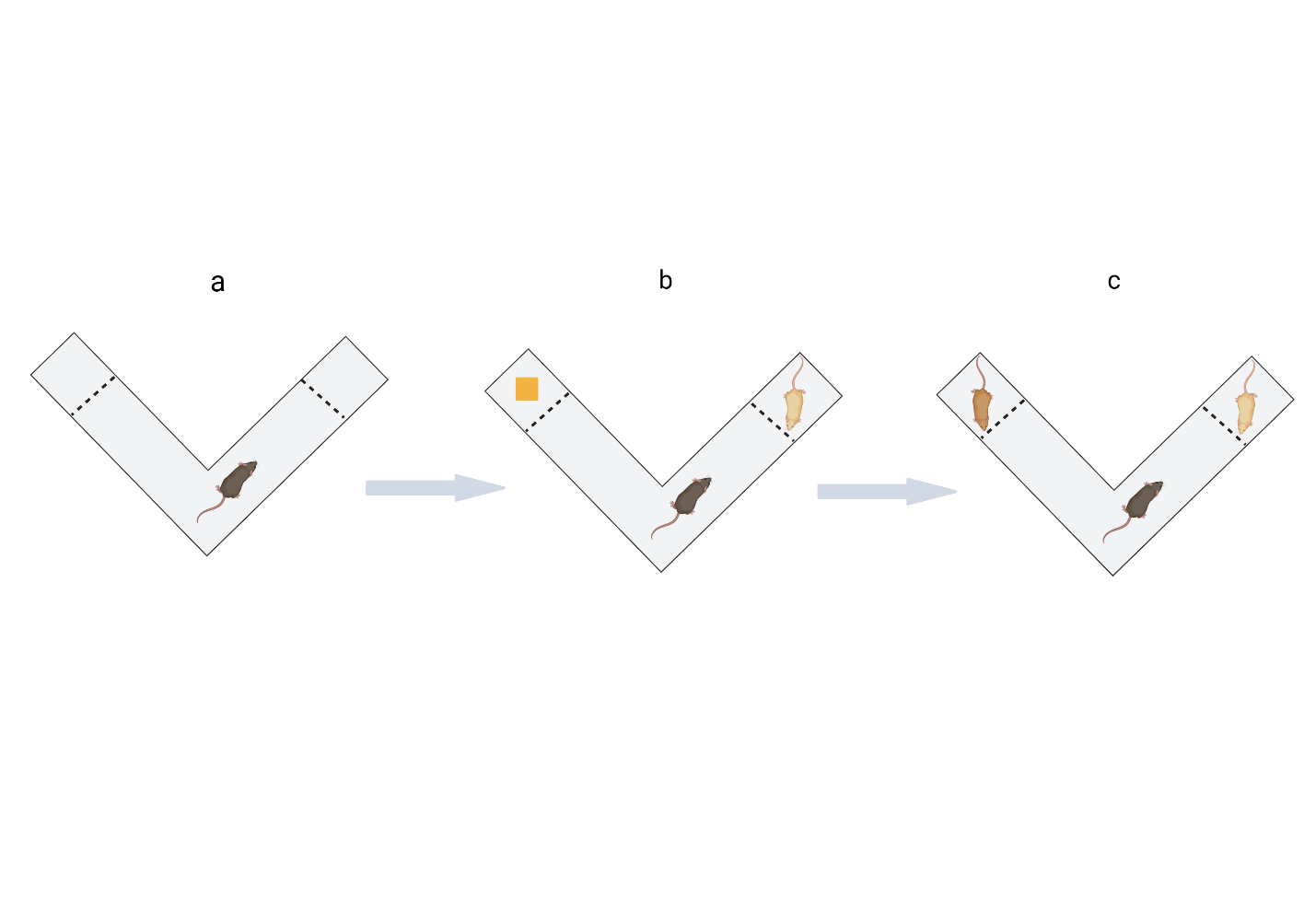


**Figure S1.7** Visual example of the social interaction test. (A) Stage 1: no objects are placed behind the barriers; (B) Stage 2: an object remains in one compartment, and a mouse is placed in the other; (C) Stage 3: the mouse introduced in Stage 2 remains, while the object is replaced by a novel mouse previously unfamiliar to the test mouse.

*1.8 Glucose Preference Test*

The glucose preference test is used to evaluate motivation, depressive-like behavior, and the ability of mice to experience pleasure. This test relies on animals’ natural preference for sweetened solutions. It is conducted in the home cages of the mice and consists of three stages:

Stage 1 (Habituation): Mice are provided with two bottles containing autoclaved water for 24 hours.

Stage 2 (Glucose Solution Presentation): After habituation, one bottle is replaced with a 10% glucose solution, while the other continues to contain water. Both bottles are weighed before being returned to the cage (glucose bottle on the left, water bottle on the right) for 24 hours under normal living conditions. After 24 hours, the bottles are weighed again.

Stage 3 (Position Switch): The positions of the glucose and water bottles are swapped, and the mice are observed for an additional 24 hours. At the end of this period, the bottles are weighed again.

Glucose preference is calculated as the percentage of glucose solution consumed relative to the total fluid intake (water plus glucose solution) using the following formula:

$$\text{Glucose Preference}\%=\frac{\text{Glucose Intake}}{\text{Glucose Intake}+\text{Water Intake}}\times100$$

Reduced consumption of sweet solutions indicates anhedonia, the inability to experience pleasure, which is commonly used as a behavioral measure of depression in rodents. A decreased preference for glucose is a key indicator of anhedonia, and rodents exhibiting this behavior typically show additional behavioral and physiological changes associated with depressive-like states (Scheggi, De Montis, & Gambarana, 2018).

**2 Metabolic and Gastrointestinal Function Tests**

*2.1* *Glucose Tolerance Test*

The glucose tolerance test assesses how efficiently glucose is metabolized. Mice undergo a 12-hour fasting period prior to testing.

In the first stage, baseline blood glucose levels are measured using the Contour Plus ELITE blood glucose monitoring system (Ascensia Diabetes Care, Switzerland) by collecting blood from the tip of the mouse’s tail.

In the second stage, glucose solution (2 mg/kg) is administered via intraperitoneal injection. Blood glucose levels are then measured at 15, 30, 45, 60, and 120 minutes post-injection.

Data analysis focuses on the rate of glucose level increase, the duration of elevated glucose levels, and whether glucose levels return to baseline within 120 minutes.

*2.2* *Gastrointestinal Transit Time Test*

The gastrointestinal transit time test evaluates the efficiency of digestive tract motility by measuring the time taken for ingested substances to pass through the system.

Mice are orally administered 0.2 ml of carmine dye, a bright red pigment derived from cochineal insects, via gastric gavage (18 gauge, 30 mm). The time of dye administration and the time of its first appearance in feces are recorded. The interval between administration and excretion is defined as the gastrointestinal transit time, which reflects gastrointestinal motility.

Impaired motility, as indicated by prolonged transit time, is commonly associated with gastrointestinal disorders (Kacmaz et al., 2021).

**3 Hematological and Immune Assays**

Hematological and immune assays were used to assess inflammation and anemia, distinguish between bacterial and viral infections, and evaluate the quantity, size, and composition of blood cellular elements.

*3.1* *Peripheral blood cell counts*

Blood samples were collected from the tip of the tail using EDTA-coated capillary tubes. A volume of 10 µL of blood was obtained per sample (one per mouse). Peripheral blood cell counts, including white blood cells (WBC), lymphocytes (LYM), monocytes (MONO), granulocytes (GRAN), red blood cell (RBC), hemoglobin (HGB) were measured using the Exigo hematology analyzer (Boule Diagnostics AB, Sweden).

*3.2 Plasma LPS Detection*

Blood samples were collected during culling into tubes containing 10 µL of EDTA (2 mg/mL). The samples were immediately placed on ice and centrifuged at 4 °C at 1500 g for 15 minutes. Plasma was then transferred into fresh vials and stored at −80 °C until analysis. Plasma lipopolysaccharide (LPS) levels were measured using the Pierce™ Chromogenic Endotoxin Quant Kit (Thermo Fisher Scientific, Waltham, MA, USA), following the manufacturer’s protocol.

**4 Targeted hippocampal gene expression analysis**

After the animals were culled, hippocampal samples were collected in 1.5 ml tubes. The samples were immediately placed on dry ice and transferred to a −80 °C freezer. Prior to gene expression analysis, the samples were kept on ice until homogenization and subsequent processing.

Total RNA was extracted from hippocampal tissue using the Invitrogen PureLink RNA Mini Kit (Thermo Fisher Scientific, Waltham, MA, USA) according to the manufacturer’s instructions.

Complementary DNA (cDNA) was synthesized from total RNA using the High-Capacity cDNA Reverse Transcription Kit (Thermo Fisher Scientific, Waltham, MA, USA) according to the manufacturer’s protocol. Reverse transcription reactions were performed on a Veriti Thermal Cycler (Thermo Fisher Scientific, Waltham, MA, USA).

Quantitative PCR was performed using SYBR Green qPCR Master Mix (Thermo Fisher Scientific, Waltham, MA, USA) according to the manufacturer’s instructions. Reactions were run on a QuantStudio 3 Real-Time PCR System (Thermo Fisher Scientific, Waltham, MA, USA). Relative gene expression levels were calculated by normalizing the expression of genes of interest to that of *Gapdh*.

**5 16S rRNA Sequencing and Processing**

*5.1 16S rRNA Gene Amplicon Library Preparation*

The V3–V4 hypervariable region of the bacterial 16S rRNA gene was amplified using dual-indexing primers as described by Fadrosh et al (Fadrosh et al., 2014). PCR reactions (27 µL total volume) contained 12.5 µL of 2× Platinum SuperFi PCR Master Mix (Invitrogen, Thermo Fisher Scientific, Waltham, MA, USA), 5 µL of 5× SuperFi GC Enhancer ((Invitrogen, Thermo Fisher Scientific, Waltham, MA, USA), 4 µL of nuclease-free water, 2 µL of forward primer (10 µM), 2 µL of reverse primer (10 µM), and 1.5 µL of template DNA. Reactions were assembled on ice with enough master mix prepared for two 96-well plates plus a 10% excess. Thermocycling was performed on an Applied Biosystems Veriti Thermal Cycler (Applied Biosystems, Thermo Fisher Scientific, Waltham, MA) under the following conditions: initial denaturation at 98 °C for 30 seconds; 30 cycles of 98 °C for 10 seconds, 55 °C for 20 seconds, and 72 °C for 30 seconds; final extension at 72 °C for 5 min

*5.2 Amplicon Verification*

Five microliters of each PCR product were mixed with 1 µL of loading dye and run on a 2% agarose gel in 1× TBE buffer containing 0.5 µg/mL ethidium bromide. Electrophoresis was performed at 150 V for 25 minutes. Successful amplification was confirmed by the presence of a single band of approximately 450 bp, visualized alongside a 100 bp DNA ladder.

*5.3 Normalization and Pooling*

PCR products were normalized using the SequalPrep Normalization Plate Kit (Invitrogen, Thermo Fisher Scientific, Waltham, MA) according to the manufacturer’s instructions. Normalized amplicons were quantified using a Qubit 3.0 fluorometer with the Qubit dsDNA HS Assay Kit (Invitrogen, Thermo Fisher Scientific, Waltham, MA). Four microliters from each well were pooled and vortexed thoroughly. The concentration of the pooled library was measured at 0.506 ng/µL, corresponding to approximately 1.7 nM, assuming an average amplicon length of 450 bp.

*5.4 Library Concentration and Final Quantification*

A 300 µL aliquot of the pooled library was concentrated to 98 µL using a vacuum concentrator at 35 °C for 40 minutes. Final quantification was performed using the Colibri™ Quantification Master Mix (Invitrogen, Thermo Fisher Scientific, Waltham, MA) with standards STD1–STD6, a no-template control, and two serial dilutions (1:10,000 and 1:100,000) of the concentrated library.

*5.5 Sequencing*

Libraries were denatured, diluted to 8 pM with a 10% PhiX control spike-in, and sequenced on an Illumina MiSeq platform using the MiSeq Reagent Kit v3 (600-cycle, paired-end) (Illumina, Inc., San Diego, CA, USA).

*5.6 Bioinformatic Processing*

Demultiplexing of dual-indexed reads was performed using Pheniqs (Galanti, Shasha, & Gunsalus, 2021), configured for the barcodes and primers described by Fadrosh et al (Fadrosh et al., 2014). Primer removal and quality trimming were carried out using cutadapt (Martin, 2011). This step removed read pairs lacking primer sequences and discarded bases with quality scores below 20.

The resulting per-sample FASTQ files, containing raw paired-end sequences, were processed using the DADA2 pipeline (Callahan et al., 2016). Forward and reverse reads were trimmed to 250 and 220 bp, respectively, and filtered with a maximum expected error of 2 and 5, respectively. Error rates were learned per read direction, and Amplicon Sequence Variants (ASVs) were inferred using the DADA algorithm. Paired-end reads were merged with a minimum 20 bp overlap and overhang trimming. Chimeras were removed using the consensus method, and only ASVs between 401 and 430 bp in length were retained.

Taxonomic assignment was performed to the genus level against the SILVA reference database (version 138.2) using a minimum bootstrap confidence threshold of 80.

*5.7 OTU-based processing and metabolic network feature generation (MetNet workflow)*

To enable metabolic network inference, a complementary OTU-based pipeline was applied to the same demultiplexed reads. Quality-filtered FASTQ files were converted to FASTA format and screened for ambiguous bases with BBDuk v39.01 (Bushnell, 2014). Reads were dereplicated (vsearch --derep_fulllength --sizeout) and clustered using VSEARCH v2.22.1–2.30.0 (Rognes, Flouri, Nichols, Quince, & Mahé, 2016) and Swarm v3.1.1 (Mahé, Rognes, Quince, de Vargas, & Dunthorn, 2014). Representative sequences underwent reference-guided chimera removal against SILVA (*vsearch --uchime_ref*), followed by de novo filtering (*--uchime3_denovo*). Swarm centroids were truncated to ≤ 420 bp before taxonomy assignment, which was performed with DADA2 v1.32.0 against SILVA v138.1. Taxonomic assignment for OTU-based clustering used SILVA v138.1 for compatibility with the q2-metnet workflow. OTU abundance tables were generated by reconciling the VSEARCH and Swarm outputs to preserve quantitative integrity for downstream analyses. The resulting OTU tables and taxonomy annotations were converted to QIIME 2 artifacts (*FeatureTable[Frequency]* and *FeatureData[Taxonomy]*) and analyzed using the q2-metnet plugin (version 1.0.2, QIIME 2 2024.10.1) (Bolyen et al., 2019). The AGREDA metabolic model was applied at the genus level via the *generate-features* action (input-interest flag enabled). This yielded reaction- and subsystem-level flux scores and a corresponding design matrix for differential network analysis.

*5.8 Differential metabolic network analysis*

To enhance stability, flux features were retained if > 0.1 in ≥ 5 % of mice, above the 10th percentile for variance and interquartile range, and within the top 5 % by coefficient of variation. Bray-Curtis PERMANOVA confirmed separation by experimental group. Fluxes were clamped to [ε, 1 − ε] (ε = 1 × 10⁻⁴) and logit-transformed before modeling.

Per-feature generalized linear mixed-effects models were fitted using glmmTMB (Brooks, Mollie et al., 2017) with the formula *logit(flux) ~ Condition + (1 | Subject)*. Pairwise contrasts (AD, T2DM, AD+T2DM vs. control) were computed with emmeans (Searle, Speed, & Milliken, 1980), and Benjamini-Hochberg correction was applied. The resulting contrasts identified reaction- and substrate-level depletions of secondary-metabolite pathways.

**References**

Bolyen, E., Rideout, J. R., Dillon, M. R., Bokulich, N. A., Abnet, C. C., Al-Ghalith, G. A., … Caporaso, J. G. (2019). Reproducible, interactive, scalable and extensible microbiome data science using QIIME 2. *Nature Biotechnology*, *37*(8), 852–857. https://doi.org/10.1038/s41587-019-0209-9

Brooks, Mollie, E., Kristensen, K., Benthem, Koen, J., V., Magnusson, A., Berg, Casper, W., Nielsen, A., … Bolker, Benjamin, M. (2017). glmmTMB Balances Speed and Flexibility Among Packages for Zero-inflated Generalized Linear Mixed Modeling. *The R Journal*, *9*(2), 378. https://doi.org/10.32614/RJ-2017-066

Bushnell, B. (2014). *BBMap: A Fast, Accurate, Splice-Aware Aligner*. Retrieved from https://escholarship.org/uc/item/1h3515gn

Callahan, B. J., McMurdie, P. J., Rosen, M. J., Han, A. W., Johnson, A. J. A., & Holmes, S. P. (2016). DADA2: High-resolution sample inference from Illumina amplicon data. *Nature Methods*, *13*(7), 581–583. https://doi.org/10.1038/nmeth.3869

Fadrosh, D. W., Ma, B., Gajer, P., Sengamalay, N., Ott, S., Brotman, R. M., & Ravel, J. (2014). An improved dual-indexing approach for multiplexed 16S rRNA gene sequencing on the Illumina MiSeq platform. *Microbiome*, *2*(1), 6. https://doi.org/10.1186/2049-2618-2-6

Galanti, L., Shasha, D., & Gunsalus, K. C. (2021). Pheniqs 2.0: accurate, high-performance Bayesian decoding and confidence estimation for combinatorial barcode indexing. *BMC Bioinformatics*, *22*(1), 359. https://doi.org/10.1186/s12859-021-04267-5

Kacmaz, H., Alto, A., Knutson, K., Linden, D. R., Gibbons, S. J., Farrugia, G., & Beyder, A. (2021). A simple automated approach to measure mouse whole gut transit. *Neurogastroenterology & Motility*, *33*(2). https://doi.org/10.1111/nmo.13994

Komada, M., Takao, K., & Miyakawa, T. (2008). Elevated Plus Maze for Mice. *Journal of Visualized Experiments*, (22). https://doi.org/10.3791/1088

Kraeuter, A.-K., Guest, P. C., & Sarnyai, Z. (2019). *The Y-Maze for Assessment of Spatial Working and Reference Memory in Mice*. https://doi.org/10.1007/978-1-4939-8994-2_10

Mahé, F., Rognes, T., Quince, C., de Vargas, C., & Dunthorn, M. (2014). Swarm: robust and fast clustering method for amplicon-based studies. *PeerJ*, *2*, e593. https://doi.org/10.7717/peerj.593

Manzanares, G., Brito-da-Silva, G., & Gandra, P. G. (2019). Voluntary wheel running: patterns and physiological effects in mice. *Brazilian Journal of Medical and Biological Research*, *52*(1). https://doi.org/10.1590/1414-431x20187830

Martin, M. (2011). Cutadapt removes adapter sequences from high-throughput sequencing reads. *EMBnet.Journal*, *17*(1), 10. https://doi.org/10.14806/ej.17.1.200

Martínez-Torres, S., Gomis-González, M., Navarro-Romero, A., Maldonado, R., & Ozaita, A. (2019). Use of the Vsoc-maze to Study Sociability and Preference for Social Novelty in Rodents. *BIO-PROTOCOL*, *9*(20). https://doi.org/10.21769/BioProtoc.3393

Rognes, T., Flouri, T., Nichols, B., Quince, C., & Mahé, F. (2016). VSEARCH: a versatile open source tool for metagenomics. *PeerJ*, *4*, e2584. https://doi.org/10.7717/peerj.2584

Scheggi, S., De Montis, M. G., & Gambarana, C. (2018). Making Sense of Rodent Models of Anhedonia. *International Journal of Neuropsychopharmacology*, *21*(11), 1049–1065. https://doi.org/10.1093/ijnp/pyy083

Searle, S. R., Speed, F. M., & Milliken, G. A. (1980). Population Marginal Means in the Linear Model: An Alternative to Least Squares Means. *The American Statistician*, *34*(4), 216–221. https://doi.org/10.1080/00031305.1980.10483031

Supplementary Table 1. General characteristics of human donors

|  | **healthy (N=8)** | **T2DM (N=9)** | **AD (N=11)** | **AD+T2DM (N=5)** | ***p* value** |
| --- | --- | --- | --- | --- | --- |
| Age (Mean ± SD) | 68.6 ± 5.7 | 69.7 ± 7.8 | 70.4 ± 8.3 | 68.4 ± 7.3 | 0.790 |
| Sample Origin:  Lithuania  Poland | 5.0 (62.5%)  3.0 (37.5%) | 5.0 (55.6%)  4.0 (44.4%) | 5.0 (45.5%)  6.0 (54.5%) | 2 (40.0%)  3 (60.0%) | 0.880 |

Data presented as mean ± SD or number (%). Differences across groups tested with one-way ANOVA or Chi-square test, as appropriate. Significant *p* < 0.05.

Supplementary Table 2. Pairwise differences in alpha diversity indices Chao1, Shannon, and Simpson across human FMT donors groups. Kruskal-Wallis tests followed by Dunn’s post-hoc comparisons (Benjamini-Hochberg adjusted) was used to identify significant differences. Significant adjusted *p* < 0.05.

| **Index** | **Comparison** | **Estimate** | ***p* value** | ***p* adj.** |
| --- | --- | --- | --- | --- |
| Chao1 | AD vs. AD+T2DM | 0.8611 | 0.3892 | 0.7618 |
|  | AD vs. healthy | 0.1872 | 0.8515 | 0.9901 |
|  | AD+T2DM vs. healthy | −0.6621 | 0.5079 | 0.7618 |
|  | AD vs. T2DM | 1.0180 | 0.3087 | 0.7618 |
|  | AD+T2DM vs. T2DM | −0.0124 | 0.9901 | 0.9901 |
|  | healthy vs. T2DM | 0.7626 | 0.4457 | 0.7618 |
| Shannon | AD vs. AD+T2DM | 0.7286 | 0.4662 | 0.8701 |
|  | AD vs. healthy | 0.1669 | 0.8674 | 0.8886 |
|  | AD+T2DM vs. healthy | −0.5533 | 0.5801 | 0.8701 |
|  | AD vs. T2DM | 1.0482 | 0.2946 | 0.8701 |
|  | AD+T2DM vs. T2DM | 0.1401 | 0.8886 | 0.8886 |
|  | healthy vs. T2DM | 0.8099 | 0.4180 | 0.8701 |
| Simpson | AD vs. AD+T2DM | 0.2894 | 0.7723 | 0.9268 |
|  | AD vs. healthy | 0.3693 | 0.7119 | 0.9268 |
|  | AD+T2DM vs. healthy | 0.0272 | 0.9783 | 0.9783 |
|  | AD vs. T2DM | 1.0784 | 0.2809 | 0.9268 |
|  | AD+T2DM vs. T2DM | 0.5892 | 0.5557 | 0.9268 |
|  | healthy vs. T2DM | 0.6444 | 0.5193 | 0.9268 |

Supplementary Table 3. PERMANOVA results obtained by testing the effects of group, age, and nationality on human gut microbiota composition (Aitchison distances). *p* values were adjusted using the Benjamini-Hochberg procedure. Significant adjusted *p* < 0.05.

|  | **Df** | **Sum of Squares** | **R²** | **F** | ***p* adj.** |
| --- | --- | --- | --- | --- | --- |
| Group | 3 | 3709.6 | 0.1222 | 1.3340 | 0.032 |
| Age | 1 | 809.0 | 0.0267 | 0.8728 | 0.644 |
| Nationality | 1 | 812.5 | 0.0268 | 0.8766 | 0.652 |
| Residual | 27 | 25026.0 | 0.8244 |  |  |
| Total | 32 | 30357.0 | 1.0000 |  |  |

Supplementary Table 4. Pairwise differences in alpha diversity indices Chao1, Shannon, and Simpson between control vs. FMT recipients. Kruskal-Wallis tests followed by Dunn’s post-hoc comparisons (Benjamini-Hochberg adjusted) was used to identify significant differences. Significant adjusted *p* < 0.05 in bold.

| **Index** | **Comparison** | **Estimate** | ***p* value** | ***p* adj.** |
| --- | --- | --- | --- | --- |
| Chao1 | control vs. AD | −1.9394 | 0.0525 | 0.1749 |
|  | control vs. AD+T2DM | −2.7960 | 0.0052 | 0.0517 |
|  | control vs. healthy | −1.8077 | 0.0706 | 0.1766 |
|  | control vs. T2DM | −2.4464 | 0.0144 | 0.0721 |
| Shannon | control vs. AD | −2.8882 | 0.0039 | **0.0216** |
|  | control vs. AD+T2DM | −2.8531 | 0.0043 | **0.0216** |
|  | control vs. healthy | −1.6751 | 0.0939 | 0.1952 |
|  | control vs. T2DM | −2.2156 | 0.0267 | 0.0891 |
| Simpson | control vs. AD | −3.2250 | 0.0013 | **0.0126** |
|  | control vs. AD+T2DM | −2.3830 | 0.0172 | 0.0859 |
|  | control vs. healthy | −2.0520 | 0.0402 | 0.1004 |
|  | control vs. T2DM | −2.2156 | 0.0267 | 0.0891 |

Supplementary Table 5. PERMANOVA results obtained by testing differences in gut microbiota composition (Aitchison distances) among mice experimental groups. *p* values were adjusted using the Benjamini-Hochberg procedure. Significant *p* < 0.05.

|  | **Df** | **Sum of Squares** | **R²** | **F** | ***p* adj.** |
| --- | --- | --- | --- | --- | --- |
| Model | 3 | 1374.1 | 0.1051 | 2.272 | 0.001 |
| Residual | 58 | 11695.0 | 0.8949 |  |  |
| Total | 61 | 13069.1 | 1.0000 |  |  |

Supplementary Table 6. Group differences in hippocampal gene expression markers, blood count, and behavioral measures among experimental mice experimental groups.

|  | **control** | **healthy** | **T2DM** | **AD** | **AD+T2DM** | ***p* adj.** | ***p* adj.*** |
| --- | --- | --- | --- | --- | --- | --- | --- |
| *Ngf* relative gene expression | 0.0006 [0.0002] | 0.0004 [0.0001] | 0.0007 [0.0003] | 0.0007 [0.0003] | 0.0005 [0.0002] | **2.0×10⁻^4^** | **0.010** |
| *Dlg*4 relative gene expression | 0.3912 [0.3899] | 0.1870 [0.0775] | 0.3637 [0.1427] | 0.3819 [0.1215] | 0.2589 [0.0685] | **0.005** | **0.047** |
| *Igf*1 relative gene expression | 0.0001 [0.0002] | 5.0×10⁻^5^ [3.0×10⁻^5^] | 0.0001 [9.0×10⁻^5^] | 0.0001 [5.0×10⁻^5^] | 6.0×10⁻^5^ [5.0×10⁻^5^] | **4.0×10⁻^6^** | **0.001** |
|  |  |  |  |  |  |  |  |
| *Fos* relative gene expression | 0.0020 [0.0016] | 0.0011 [0.0010] | 0.0020 [0.0018] | 0.0026 [0.0009] | 0.0014 [0.0009] | **3.0×10⁻^4^** | **0.003** |
| *Arc* relative gene expression | 0.0380 [0.0207] | 0.0292 [0.0110] | 0.0397 [0.0277] | 0.0496 [0.0069] | 0.0241 [0.0118] | **2.2×10⁻^5^** | **0.001** |
| *Creb*1 relative gene expression | 0.0085 ± 0.0025 | 0.0042 ± 0.0017 | 0.0099 ± 0.0030 | 0.0104 ± 0.0019 | 0.0052 ± 0.0016 | **4.9×10⁻^10^** | **2.2×10⁻^10^** |
| MONO blood count (10^9^/L) | 0.7 [0.1] | 0.6 [0.2] | 0.6 [0.2] | 0.7 [0.2] | 0.6 [0.1] | 0.270 | 0.088 |
| LYM blood count (10^9^/L) | 20.2 [2.5] | 18.7 [3.2] | 16.3 [5.4] | 16.7 [2.4] | 16.4 [1.3] | **0.010** | 0.500 |
| WBC blood count (10^9^/L) | 25.6 [4.2] | 24.3 [5.1] | 21.1 [6.2] | 23.3 [4.2] | 20.6 [1.4] | **0.003** | **0.037** |
| GRAN blood count (10^9^/L) | 5.0 [1.9] | 5.4 [1.3] | 4.0 [1.0] | 5.2 [1.4] | 3.8 [0.9] | **0.002** | **0.001** |
| HGB blood count (10^9^/L) | 14.5 ± 0.7 | 14.3 ± 1.2 | 14.0 ± 0.8 | 14.1 ± 0.8 | 14.3 ± 0.7 | 0.840 | 0.850 |
| RBC blood count (10^9^/L) | 8.9 [0.6] | 8.9 [0.9] | 8.8 [0.8] | 9.2 [0.8] | 9.0 [0.4] | 0.960 | 0.790 |
| PM open space time (s) | 48.0 [32.0] | 56.0 [43.5] | 43.0 [37.0] | 33.5 [59.5] | 65.5 [32.5] | 0.570 | 0.430 |
| PM open arm end time (s) | 11.5 [17.2] | 11.0 [20.5] | 9.0 [19.0] | 2.5 [12.5] | 14.5 [17.8] | 0.620 | 0.510 |
| PM No. of times at open arm end | 2.5 [4.0] | 1.0 [3.0] | 1.0 [2.0] | 0.5 [2.2] | 2.5 [2.5] | 0.530 | 0.350 |
| PM No. of times crossing center | 0.5 [1.0] | 1.0 [3.0] | 3.0 [3.0] | 1.0 [3.0] | 2.0 [1.0] | 0.170 | 0.330 |
| PM No. of times entering open space | 8.5 ± 3.7 | 10.8 ± 3.5 | 7.3 ± 3.3 | 7.2 ± 4.2 | 10.1 ± 2.5 | 0.270 | 0.089 |
| BM primary latency (s) | 30.0 [21.2] | 57.0 [74.0] | 60.0 [55.0] | 34.0 [49.5] | 33.5 [54.2] | 0.700 | 0.780 |
| BM total latency (s) | 41.5 [29.0] | 46.0 [88.5] | 94.0 [69.0] | 43.0 [105.2] | 37.0 [61.2] | 0.650 | 0.510 |
| BM No. of primary errors | 6.0 [5.0] | 8.0 [4.0] | 14.0 [13.0] | 5.5 [11.5] | 5.5 [14.5] | 0.570 | 0.510 |
| BM No. of total errors | 6.0 [2.0] | 14.0 [12.5] | 17.0 [14.0] | 18.0 [15.2] | 12.5 [13.0] | 0.410 | 0.510 |
| YM No. of visited arms | 24.8 ± 8.6 | 18.7 ± 4.3 | 15.2 ± 6.6 | 18.6 ± 9.5 | 17.4 ± 6.5 | 0.370 | 0.850 |
| YM confirmation number | 15.3 ± 7.1 | 11.2 ± 2.5 | 9.2 ± 4.8 | 11.2 ± 5.8 | 10.4 ± 4.2 | 0.370 | 0.850 |
| YM % | 65.2 ± 13.8 | 68.3 ± 11.1 | 69.5 ± 9.6 | 71.0 ± 12.6 | 68.6 ± 9.6 | 0.890 | 0.900 |
| YM average velocity (cm/s) | 4.9 ± 1.2 | 3.9 ± 0.6 | 3.5 ± 1.0 | 3.9 ± 1.4 | 3.8 ± 0.9 | 0.370 | 0.850 |
| YM track length (cm) | 2655.6 ± 618.9 | 2113.3 ± 340.2 | 1873.4 ± 522.5 | 2088.3 ± 760.7 | 2051.2 ± 492.1 | 0.370 | 0.850 |
| YM activity % | 52.1 ± 10.2 | 42.6 ± 6.7 | 39.3 ± 10.3 | 43.1 ± 11.4 | 41.2 ± 8.7 | 0.370 | 0.850 |
| NOR DI | -0.0 ± 0.2 | 0.2 ± 0.3 | 0.1 ± 0.3 | 0.1 ± 0.3 | 0.1 ± 0.2 | 0.810 | 0.850 |
| OF total average velocity (cm/s) | 4.5 [0.9] | 5.6 [2.5] | 5.3 [1.1] | 5.8 [2.3] | 5.1 [1.1] | 0.710 | 0.800 |
| OF total track length (cm) | 2871.5 ± 682.4 | 3446.8 ± 918.6 | 3285.2 ± 730.2 | 3366.4 ± 872.0 | 3046.2 ± 514.3 | 0.720 | 0.850 |
| OF total activity % | 46.6 ± 8.0 | 52.5 ± 9.5 | 51.8 ± 7.3 | 52.6 ± 9.5 | 49.1 ± 5.0 | 0.720 | 0.850 |
| OF No. of total ambulations | 2118.5 ± 102.2 | 2110.0 ± 164.3 | 2172.8 ± 107.4 | 2161.8 ± 175.8 | 2171.1 ± 122.6 | 0.810 | 0.850 |
| OF No. of total zone crossing | 208.8 ± 63.8 | 291.4 ± 78.9 | 263.2 ± 74.8 | 287.1 ± 93.2 | 240.6 ± 37.4 | 0.370 | 0.850 |
| OF No. of total rearing zone crossing | 497.0 [160.8] | 718.0 [360.5] | 595.0 [283.0] | 689.0 [402.2] | 524.0 [165.0] | 0.570 | 0.560 |
| OF big zone duration (s) | 563.1 [13.0] | 550.7 [21.8] | 553.7 [34.1] | 558.5 [23.6] | 569.0 [22.9] | 0.370 | 0.350 |
| OF big zone velocity (cm/s) | 4.5 ± 1.1 | 5.5 ± 1.5 | 5.2 ± 1.1 | 5.3 ± 1.4 | 4.8 ± 0.8 | 0.720 | 0.850 |
| OF big zone track length (cm) | 2560.8 ± 579.3 | 3003.8 ± 800.7 | 2841.7 ± 604.6 | 2947.4 ± 711.9 | 2735.2 ± 469.9 | 0.800 | 0.850 |
| OF No. of big zone visits | 21.5 [14.2] | 36.0 [29.5] | 34.0 [18.0] | 32.0 [13.8] | 28.5 [16.2] | 0.630 | 0.760 |
| OF big zone activity % | 45.2 ± 8.0 | 51.3 ± 9.7 | 50.5 ± 7.2 | 51.2 ± 9.5 | 48.0 ± 5.0 | 0.720 | 0.850 |
| OF No. of big zone ambulations | 2024.3 ± 83.2 | 1957.7 ± 149.2 | 2009.5 ± 151.3 | 2041.3 ± 161.9 | 2067.6 ± 136.6 | 0.720 | 0.850 |
| OF big zone visit latency (s) | 0.0 [0.0] | 0.0 [0.0] | 0.0 [0.0] | 0.0 [0.0] | 0.0 [0.9] | 0.600 | 0.420 |
| OF small zone duration (s) | 32.4 [18.0] | 42.9 [18.6] | 39.9 [33.3] | 38.0 [23.2] | 31.0 [15.8] | 0.270 | 0.330 |
| OF small zone velocity (cm/s) | 10.9 ± 2.1 | 9.8 ± 2.5 | 10.2 ± 3.8 | 10.4 ± 2.6 | 10.9 ± 2.9 | 0.890 | 0.870 |
| OF small zone track length (cm) | 359.1 [131.4] | 380.9 [96.9] | 355.2 [174.2] | 417.8 [214.0] | 240.9 [180.5] | 0.270 | 0.330 |
| OF No. of small zone visits | 19.0 [5.0] | 26.0 [11.5] | 23.0 [14.0] | 31.0 [17.2] | 16.5 [10.0] | 0.270 | 0.330 |
| OF small zone activity % | 76.2 [4.4] | 68.8 [12.4] | 73.5 [18.9] | 74.1 [9.8] | 76.4 [13.7] | 0.700 | 0.850 |
| OF No. of small zone ambulations | 82.5 [63.2] | 111.0 [77.0] | 127.0 [102.0] | 106.5 [70.0] | 66.0 [60.5] | 0.410 | 0.380 |
| OF small zone visit latency (s) | 10.0 [14.3] | 7.8 [20.4] | 15.1 [22.3] | 8.9 [47.1] | 11.4 [36.7] | 0.950 | 0.840 |
| Running wheel daytime 12h (rev) | 782.5 [541.5] | 768.0 [319.5] | 1979.0 [1704.0] | 3599.5 [2904.2] | 956.5 [1496.2] | **0.007** | 0.280 |
| Running wheel nighttime 12h (rev) | 9664.0 [1265.0] | 11188.0 [3415.5] | 10249.0 [3774.0] | 9041.0 [8502.2] | 12239.5 [1443.0] | 0.410 | 0.380 |
| Running wheel day/night 24h (rev) | 9957.5 ± 1659.3 | 11360.9 ± 2667.7 | 10225.9 ± 3284.1 | 10809.9 ± 2607.7 | 12060.0 ± 2366.8 | 0.720 | 0.850 |
| Grooming time (s) | 15.5 [8.2] | 13.0 [8.0] | 10.0 [5.0] | 16.0 [8.5] | 10.0 [11.8] | 0.250 | 0.250 |
| Short term memory DI | 0.2 ± 0.2 | 0.3 ± 0.2 | 0.2 ± 0.2 | 0.3 ± 0.2 | 0.4 ± 0.2 | 0.370 | 0.410 |
| Sniffing test DI | 0.7 [0.1] | 0.7 [0.3] | 0.5 [0.4] | 0.3 [0.3] | 0.6 [0.1] | **0.007** | **0.042** |
| Social maze social ability index | 0.3 ± 0.2 | 0.3 ± 0.2 | 0.3 ± 0.2 | 0.4 ± 0.2 | 0.2 ± 0.2 | 0.800 | 0.850 |
| Social maze social novelty index | 0.2 ± 0.2 | 0.2 ± 0.1 | 0.2 ± 0.3 | 0.1 ± 0.2 | 0.2 ± 0.1 | 0.720 | 0.850 |
| GPT % of sugar from fluid | 59.3 [41.1] | 85.6 [12.5] | 77.8 [11.7] | 79.2 [8.9] | 78.4 [5.8] | 0.560 | 0.930 |
| GPT DI | 0.2 [0.8] | 0.7 [0.3] | 0.6 [0.2] | 0.6 [0.2] | 0.6 [0.1] | 0.560 | 0.930 |
| GTT basal (mg/dL) | 5.2 [0.5] | 5.2 [1.0] | 5.1 [0.7] | 4.9 [0.8] | 5.3 [0.5] | 0.370 | 0.220 |
| GTT 15 min (mg/dL) | 21.8 [1.0] | 19.7 [8.7] | 19.2 [10.7] | 20.7 [2.6] | 20.0 [1.9] | 0.860 | 0.930 |
| GTT 30 min (mg/dL) | 23.2 [1.4] | 22.2 [10.5] | 23.6 [12.8] | 22.0 [3.6] | 23.6 [7.2] | 0.860 | 0.930 |
| GTT 45 min (mg/dL) | 18.0 [1.3] | 15.2 [7.9] | 18.4 [11.2] | 17.7 [4.2] | 18.2 [7.0] | 0.690 | 0.930 |
| GTT 60 min (mg/dL) | 13.1 [0.9] | 11.6 [4.1] | 13.6 [6.5] | 12.8 [3.1] | 13.4 [4.3] | 0.560 | 0.930 |
| GTT 120 min (mg/dL) | 7.0 [1.1] | 6.8 [0.8] | 6.3 [1.4] | 6.8 [0.8] | 6.8 [1.2] | 0.690 | 0.790 |
| Carmine test transit time (s) | 92.0 [19.5] | 74.0 [19.5] | 86.0 [24.0] | 85.5 [14.2] | 99.0 [45.5] | 0.560 | 0.930 |
| Cecum weight (g) | 0.6 [0.1] | 0.8 [0.2] | 0.8 [0.3] | 0.9 [0.2] | 0.8 [0.1] | **0.019** | 0.220 |
| Plasma LPS (eu/ml) | 10.9 [2.6] | 10.5 [1.6] | 10.6 [0.4] | 11.3 [1.3] | 10.8 [1.0] | 0.510 | 0.220 |
| Average weight (g) | 25.1 ± 1.4 | 25.5 ± 1.4 | 25.7 ± 1.6 | 25.8 ± 1.4 | 24.9 ± 1.3 | 0.720 | 0.850 |

MONO, monocytes; LYM, lymphocytes; WBC, white blood cells; GRAN, granulocytes; HGB, hemoglobin; RBC, red blood cells; PM, plus maze; YM, Y maze; NOR, novel object recognition; OF, open field; DI discrimination index; GPT, glucose preference test; GTT, glucose tolerance test; LPS, lipopolysaccharide. Data are presented as mean ± SD or median [IQR]. Differences across groups were tested using one-way ANOVA or Kruskal-Wallis test, as appropriate. p adj. = overall comparison between control and FMT groups. *p* adj.* = comparison among FMT groups. Significant p < 0.05 in bold.

Supplementary Table 7. Pairwise comparison in hippocampal gene expression markers, blood count, and behavioral measures between mice control group vs. FMT recipients.

| **Variable** | **Comparison** | **Estimate** | ***p* adj.** |
| --- | --- | --- | --- |
| *Ngf* relative gene expression | control vs. AD | −0.0001 | 0.3862 |
|  | control vs. AD+T2DM | 0.0001 | 0.3336 |
|  | control vs. healthy | 0.0002 | 0.0916 |
|  | control vs. T2DM | −0.0001 | 0.6193 |
| *Dlg*4 relative gene expression | control vs. AD | 0.0093 | 0.9607 |
|  | control vs. AD+T2DM | 0.1324 | 0.2780 |
|  | control vs. healthy | 0.2043 | 0.0747 |
|  | control vs. T2DM | 0.0275 | 0.9009 |
| *Igf*1 relative gene expression | control vs. AD | −3.12×10⁻^5^ | 0.4092 |
|  | control vs. AD+T2DM | 3.47×10⁻^5^ | 0.1991 |
|  | control vs. healthy | 4.30×10⁻^5^ | 0.0530 |
|  | control vs. T2DM | −4.44×10⁻^5^ | 0.3034 |
| *Fos* relative gene expression | control vs. AD | −0.0006 | 0.4990 |
|  | control vs. AD+T2DM | 0.0006 | 0.1450 |
|  | control vs. healthy | 0.0009 | 0.0678 |
|  | control vs. T2DM | 3.19×10⁻^5^ | 0.6589 |
| *Arc* relative gene expression | control vs. AD | −0.0115 | 0.3592 |
|  | control vs. AD+T2DM | 0.0139 | 0.0605 |
|  | control vs. healthy | 0.0088 | 0.0548 |
|  | control vs. T2DM | −0.0017 | 0.8687 |
| *Creb1* relative gene expression | control vs. AD | −0.0018 | 0.4034 |
|  | control vs. AD+T2DM | 0.0033 | **0.0413** |
|  | control vs. healthy | 0.0042 | **0.0023** |
|  | control vs. T2DM | −0.0014 | 0.7013 |
| LYM (10⁹/L) | control vs AD | 3.5500 | 0.0844 |
|  | control vs. AD+T2DM | 3.9000 | 0.0699 |
|  | control vs. healthy | 1.5500 | 0.5995 |
|  | control vs. T2DM | 3.9500 | **0.0150** |
| WBC (10⁹/L) | control vs. AD | 2.3000 | 0.4868 |
|  | control vs. AD+T2DM | 4.9500 | **0.0314** |
|  | control vs. healthy | 1.3000 | 0.8327 |
|  | control vs. T2DM | 4.5000 | **0.0267** |
| GRAN (10⁹/L) | control vs. AD | −0.1500 | 0.4155 |
|  | control vs. AD+T2DM | 1.2500 | 0.1611 |
|  | control vs. healthy | −0.4000 | 0.5872 |
|  | control vs. T2DM | 1.0000 | 0.3128 |
| Running wheel daytime (12h) (rev) | control vs. AD | −2817.0 | **0.0222** |
|  | control vs. AD+T2DM | −174.0 | 0.4445 |
|  | control vs. healthy | 14.5 | 0.7614 |
|  | control vs. T2DM | −1196.5 | 0.1959 |
| Sniffing test DI | control vs. AD | 0.3961 | **0.0201** |
|  | control vs. AD+T2DM | 0.0571 | 0.9077 |
|  | control vs. healthy | −0.0519 | 0.9419 |
|  | control vs. T2DM | 0.1459 | 0.5185 |
| Cecum weight (g) | control vs. AD | −0.3458 | **0.0007** |
|  | control vs. AD+T2DM | −0.2092 | 0.1042 |
|  | control vs. healthy | −0.2052 | **0.0424** |
|  | control vs. T2DM | −0.2348 | **0.0166** |

LYM, lymphocytes; WBC, white blood cells; GRAN, granulocytes; DI, discrimination index. Post-hoc pairwise comparisons were performed only when the omnibus test was significant, using Tukey’s HSD and Dunn’s test, as appropriate. Significant differences (Benjamini-Hochberg adjusted p < 0.05) are indicated in bold.

Supplementary Table 8. Pairwise differences in alpha diversity indices Chao1, Shannon, and Simpson across FMT recipients. Kruskal-Wallis tests followed by Dunn’s post-hoc comparisons (Benjamini-Hochberg adjusted) was used to identify significant differences. Significant adjusted *p* < 0.05.

| **Variable** | **Comparison** | **Estimate** | ***p* value** | ***p* adj.** |
| --- | --- | --- | --- | --- |
| Chao1 | AD vs. AD+T2DM | −1.3095 | 0.1904 | 0.5711 |
|  | AD vs. healthy | 0.1136 | 0.9096 | 0.9096 |
|  | AD+T2DM vs. healthy | 1.3373 | 0.1811 | 0.5711 |
|  | AD vs. T2DM | −0.7314 | 0.4645 | 0.6056 |
|  | AD+T2DM vs. T2DM | 0.6672 | 0.5047 | 0.6056 |
|  | healthy vs. T2DM | −0.7906 | 0.4292 | 0.6056 |
| Shannon | AD vs. AD+T2DM | −0.3578 | 0.7205 | 0.7205 |
|  | AD vs. healthy | 1.5795 | 0.1142 | 0.3427 |
|  | AD+T2DM vs. healthy | 1.6609 | 0.0967 | 0.3427 |
|  | AD vs. T2DM | 0.8263 | 0.4087 | 0.5414 |
|  | AD+T2DM vs. T2DM | 1.0317 | 0.3022 | 0.5414 |
|  | healthy vs. T2DM | −0.7535 | 0.4512 | 0.5414 |
| Simpson | AD vs. AD+T2DM | 0.7370 | 0.4611 | 0.8655 |
|  | AD vs. healthy | 1.4199 | 0.1556 | 0.5929 |
|  | AD+T2DM vs. healthy | 0.4888 | 0.6250 | 0.8655 |
|  | AD vs. T2DM | 1.2883 | 0.1976 | 0.5929 |
|  | AD+T2DM vs. T2DM | 0.3502 | 0.7262 | 0.8655 |
|  | healthy vs. T2DM | −0.1694 | 0.8655 | 0.8655 |

Supplementary Table 9. PERMANOVA results obtained by testing the effects of group, age, and nationality on FMT donors gut microbiota composition (Aitchison distances). *p* values were adjusted using the Benjamini-Hochberg procedure. Significant adjusted *p* < 0.05.

|  | **Df** | **Sum of Squares** | **R²** | **F** | ***p* value** |
| --- | --- | --- | --- | --- | --- |
| Group | 3 | 1374.1 | 0.1051 | 2.3196 | 0.001 |
| Donor age | 1 | 369.8 | 0.0283 | 1.8726 | 0.006 |
| Donor nationality | 1 | 266.9 | 0.0204 | 1.3516 | 0.110 |
| Residual | 56 | 11058.3 | 0.8461 |  |  |
| Total | 61 | 13069.1 | 1.0000 |  |  |

Supplementary Table 10. Pairwise comparison in hippocampal gene expression markers, blood count, and behavioral measures across mice FMT groups.

| **Variable** | **Comparison** | **Estimate** | ***p* adj.** |
| --- | --- | --- | --- |
| *Ngf* relative gene expression | AD vs. AD+T2DM | 0.0002 | **0.0056** |
|  | AD vs. healthy | 0.0003 | **0.0002** |
|  | AD+T2DM vs. healthy | 0.0001 | 0.4521 |
|  | AD vs. T2DM | −4.70×10⁻^7^ | 0.4857 |
|  | AD+T2DM vs. T2DM | −0.0002 | **0.0404** |
|  | healthy vs. T2DM | −0.0003 | **0.0034** |
| *Igf*1 relative gene expression | AD vs. AD+T2DM | 0.0001 | **0.0012** |
|  | AD vs. healthy | 0.0001 | **1.98×10⁻^5^** |
|  | AD+T2DM vs. healthy | 8.32×10⁻^6^ | 0.6135 |
|  | AD vs. T2DM | −1.33×10⁻^5^ | 0.6560 |
|  | AD+T2DM vs. T2DM | −0.0001 | **0.0006** |
|  | healthy vs. T2DM | −0.0001 | **1.17×10⁻^5^** |
| *Fos* relative gene expression | AD vs. AD+T2DM | 0.0012 | **0.0028** |
|  | AD vs. healthy | 0.0015 | **0.0002** |
|  | AD+T2DM vs. healthy | 0.0004 | 0.6315 |
|  | AD vs. T2DM | 0.0006 | 0.0805 |
|  | AD+T2DM vs. T2DM | −0.0006 | 0.1260 |
|  | healthy vs. T2DM | −0.0009 | **0.0428** |
| *Arc* relative gene expression | AD vs. AD+T2DM | 0.0254 | **0.0001** |
|  | AD vs. healthy | 0.0203 | **0.0001** |
|  | AD+T2DM vs. healthy | −0.0051 | 0.8596 |
|  | AD vs. T2DM | 0.0098 | 0.1055 |
|  | AD+T2DM vs. T2DM | −0.0156 | **0.0134** |
|  | healthy vs. T2DM | −0.0105 | **0.0145** |
| *Creb*1 relative gene expression | AD vs. AD+T2DM | 0.0052 | **7.59×10⁻^7^** |
|  | AD vs. healthy | 0.0061 | **7.04×10^−10^** |
|  | AD+T2DM vs. healthy | 0.0005 | 0.9163 |
|  | AD vs. T2DM | 0.0009 | 0.7631 |
|  | AD+T2DM vs. T2DM | −0.0047 | **1.04×10⁻^5^** |
|  | healthy vs. T2DM | −0.0056 | **1.89×10⁻^8^** |
| WBC (10⁹/L) | AD vs. AD+T2DM | 2.6500 | **0.0477** |
|  | AD vs. healthy | −1.0000 | 0.4802 |
|  | AD+T2DM vs. healthy | −3.6500 | **0.0184** |
|  | AD vs. T2DM | 2.2000 | **0.0144** |
|  | AD+T2DM vs. T2DM | −0.4500 | 0.8903 |
|  | healthy vs. T2DM | 3.2000 | **0.0055** |
| GRAN 10⁹/L | AD vs. AD+T2DM | 1.4000 | **0.0013** |
|  | AD vs. healthy | −0.2500 | 0.6699 |
|  | AD+T2DM vs. healthy | −1.6500 | **0.0033** |
|  | AD vs. T2DM | 1.1500 | **0.0017** |
|  | AD+T2DM vs. T2DM | −0.2500 | 0.5598 |
|  | healthy vs. T2DM | 1.4000 | **0.0075** |
| Sniffing test DI | AD vs. AD+T2DM | −0.3390 | **0.0045** |
|  | AD vs. healthy | −0.4481 | **0.0006** |
|  | AD+T2DM vs. healthy | −0.1091 | 0.7981 |
|  | AD vs. T2DM | −0.2502 | **0.0228** |
|  | AD+T2DM vs. T2DM | 0.0888 | 0.3862 |
|  | healthy vs. T2DM | 0.1979 | 0.2381 |

WBC, white blood cells; GRAN, granulocytes; DI, discrimination index. Post-hoc pairwise comparisons were performed only when the omnibus test was significant, using Tukey’s HSD and Dunn’s test, as appropriate. Significant differences (Benjamini-Hochberg adjusted p < 0.05) are indicated in bold

Supplementary Table 11. Results of Spearman correlations between differentially abundant taxa across FMT groups and host hippocampal gene expression markers that also showed significant group-level differences. Statistical significance was adjusted for multiple testing using the false discovery rate (FDR) method. Correlations exceeding |r| > 0.4 with FDR-adjusted *p* values < 0.05 were considered significant (in bold).

| **Taxon** | **Gene** | **Correlation** | ***p* value** | ***p* adj.** |
| --- | --- | --- | --- | --- |
| Acetatifactor | Creb1 | −0.1906 | 0.1409 | 0.4350 |
| Acetatifactor | Ngf | −0.1419 | 0.2786 | 0.6155 |
| Acetatifactor | Igf1 | −0.1030 | 0.4285 | 0.7251 |
| Acetatifactor | Fos | 0.0987 | 0.4519 | 0.7251 |
| Acetatifactor | Dlg4 | −0.0192 | 0.8852 | 0.9968 |
| Acetatifactor | Arc | −0.0207 | 0.8738 | 0.9968 |
| **Agathobaculum** | **Igf1** | **−0.5097** | **3.53E-05** | **0.0024** |
| **Agathobaculum** | **Creb1** | **−0.4380** | **0.0005** | **0.0217** |
| Agathobaculum | Dlg4 | −0.3150 | 0.0154 | 0.1183 |
| Agathobaculum | Arc | −0.2471 | 0.0551 | 0.2672 |
| Agathobaculum | Ngf | −0.1827 | 0.1621 | 0.4565 |
| Agathobaculum | Fos | −0.0879 | 0.5034 | 0.7634 |
| Alistipes | Fos | 0.1392 | 0.2880 | 0.6209 |
| Alistipes | Igf1 | −0.0979 | 0.4517 | 0.7251 |
| Alistipes | Ngf | −0.0378 | 0.7735 | 0.9531 |
| Alistipes | Arc | 0.0332 | 0.7990 | 0.9672 |
| Alistipes | Dlg4 | −0.0306 | 0.8178 | 0.9814 |
| Alistipes | Creb1 | 0.0172 | 0.8949 | 0.9968 |
| Anaeroplasma | Ngf | 0.3128 | 0.0153 | 0.1183 |
| Anaeroplasma | Creb1 | 0.2964 | 0.0207 | 0.1362 |
| Anaeroplasma | Dlg4 | 0.2805 | 0.0318 | 0.1826 |
| Anaeroplasma | Arc | 0.2460 | 0.0562 | 0.2672 |
| Anaeroplasma | Igf1 | 0.1954 | 0.1311 | 0.4350 |
| Anaeroplasma | Fos | 0.1849 | 0.1568 | 0.4509 |
| Anaerovoracaceae__Family_XIII_UCG-001 | Dlg4 | 0.2857 | 0.0286 | 0.1796 |
| Anaerovoracaceae__Family_XIII_UCG-001 | Ngf | 0.1093 | 0.4050 | 0.7251 |
| Anaerovoracaceae__Family_XIII_UCG-001 | Igf1 | −0.0179 | 0.8911 | 0.9968 |
| Anaerovoracaceae__Family_XIII_UCG-001 | Fos | 0.0006 | 0.9968 | 0.9968 |
| Anaerovoracaceae__Family_XIII_UCG-001 | Arc | 0.0120 | 0.9271 | 0.9968 |
| Anaerovoracaceae__Family_XIII_UCG-001 | Creb1 | 0.0080 | 0.9510 | 0.9968 |
| Anaerovoracaceae__unknown | Creb1 | 0.2426 | 0.0598 | 0.2672 |
| Anaerovoracaceae__unknown | Igf1 | 0.2403 | 0.0623 | 0.2685 |
| Anaerovoracaceae__unknown | Arc | 0.2181 | 0.0914 | 0.3602 |
| Anaerovoracaceae__unknown | Fos | 0.1497 | 0.2530 | 0.5898 |
| Anaerovoracaceae__unknown | Dlg4 | 0.1027 | 0.4377 | 0.7251 |
| Anaerovoracaceae__unknown | Ngf | 0.0552 | 0.6743 | 0.8779 |
| Bacteroides | Dlg4 | −0.2423 | 0.0646 | 0.2703 |
| Bacteroides | Igf1 | −0.1992 | 0.1237 | 0.4283 |
| Bacteroides | Fos | −0.1650 | 0.2070 | 0.5391 |
| Bacteroides | Arc | −0.1494 | 0.2496 | 0.5898 |
| Bacteroides | Creb1 | −0.1282 | 0.3240 | 0.6774 |
| Bacteroides | Ngf | −0.0565 | 0.6675 | 0.8773 |
| Barnesiella | Ngf | −0.2754 | 0.0336 | 0.1852 |
| Barnesiella | Dlg4 | −0.1772 | 0.1789 | 0.4939 |
| Barnesiella | Arc | 0.0655 | 0.6153 | 0.8407 |
| Barnesiella | Igf1 | −0.0503 | 0.6996 | 0.8938 |
| Barnesiella | Fos | 0.0481 | 0.7147 | 0.8966 |
| Barnesiella | Creb1 | −0.0085 | 0.9484 | 0.9968 |
| **Butyricimonas** | **Ngf** | **−0.5245** | **2.26E-05** | **0.0024** |
| Butyricimonas | Igf1 | −0.3779 | 0.0028 | 0.0525 |
| Butyricimonas | Creb1 | −0.3672 | 0.0038 | 0.0583 |
| Butyricimonas | Arc | −0.3474 | 0.0063 | 0.0729 |
| Butyricimonas | Fos | −0.3254 | 0.0115 | 0.1057 |
| Butyricimonas | Dlg4 | −0.2465 | 0.0600 | 0.2672 |
| Candidatus_Saccharimonas | Ngf | −0.2547 | 0.0498 | 0.2544 |
| Candidatus_Saccharimonas | Creb1 | −0.1258 | 0.3331 | 0.6813 |
| Candidatus_Saccharimonas | Igf1 | −0.1215 | 0.3499 | 0.6998 |
| Candidatus_Saccharimonas | Arc | −0.1200 | 0.3559 | 0.7015 |
| Candidatus_Saccharimonas | Dlg4 | −0.0875 | 0.5089 | 0.7634 |
| Candidatus_Saccharimonas | Fos | −0.0825 | 0.5298 | 0.7861 |
| Clostridia_UCG-014__unknown | Dlg4 | −0.1519 | 0.2501 | 0.5898 |
| Clostridia_UCG-014__unknown | Igf1 | −0.1448 | 0.2648 | 0.5991 |
| Clostridia_UCG-014__unknown | Ngf | −0.1052 | 0.4228 | 0.7251 |
| Clostridia_UCG-014__unknown | Arc | 0.0505 | 0.6984 | 0.8938 |
| Clostridia_UCG-014__unknown | Fos | −0.0223 | 0.8652 | 0.9968 |
| Clostridia_UCG-014__unknown | Creb1 | 0.0228 | 0.8613 | 0.9968 |
| Desulfovibrio | Ngf | −0.1020 | 0.4369 | 0.7251 |
| Desulfovibrio | Dlg4 | 0.1164 | 0.3791 | 0.7251 |
| Desulfovibrio | Fos | 0.0806 | 0.5393 | 0.7917 |
| Desulfovibrio | Igf1 | 0.0125 | 0.9239 | 0.9968 |
| Desulfovibrio | Arc | 0.0212 | 0.8709 | 0.9968 |
| Desulfovibrio | Creb1 | −0.0031 | 0.9811 | 0.9968 |
| Eubacterium_coprostanoligenes_group__unknown | Dlg4 | −0.1943 | 0.1400 | 0.4350 |
| Eubacterium_coprostanoligenes_group__unknown | Ngf | −0.1859 | 0.1547 | 0.4509 |
| Eubacterium_coprostanoligenes_group__unknown | Arc | 0.1604 | 0.2163 | 0.5528 |
| Eubacterium_coprostanoligenes_group__unknown | Fos | 0.1262 | 0.3357 | 0.6813 |
| Eubacterium_coprostanoligenes_group__unknown | Creb1 | 0.1035 | 0.4264 | 0.7251 |
| Eubacterium_coprostanoligenes_group__unknown | Igf1 | −0.0561 | 0.6671 | 0.8773 |
| Eubacterium_siraeum_group | Arc | 0.2071 | 0.1092 | 0.3967 |
| Eubacterium_siraeum_group | Creb1 | 0.1863 | 0.1503 | 0.4508 |
| Eubacterium_siraeum_group | Dlg4 | 0.1683 | 0.2020 | 0.5361 |
| Eubacterium_siraeum_group | Fos | 0.1557 | 0.2343 | 0.5880 |
| Eubacterium_siraeum_group | Igf1 | 0.0492 | 0.7060 | 0.8938 |
| Eubacterium_siraeum_group | Ngf | 0.0097 | 0.9412 | 0.9968 |
| Eubacterium_xylanophilum_group | Dlg4 | −0.1747 | 0.1853 | 0.5013 |
| Eubacterium_xylanophilum_group | Arc | 0.1031 | 0.4280 | 0.7251 |
| Eubacterium_xylanophilum_group | Fos | 0.0749 | 0.5685 | 0.8006 |
| Eubacterium_xylanophilum_group | Ngf | 0.0107 | 0.9351 | 0.9968 |
| Eubacterium_xylanophilum_group | Igf1 | −0.0070 | 0.9575 | 0.9968 |
| Eubacterium_xylanophilum_group | Creb1 | −0.0216 | 0.8687 | 0.9968 |
| Intestinimonas | Ngf | −0.1923 | 0.1408 | 0.4350 |
| Intestinimonas | Dlg4 | −0.1162 | 0.3796 | 0.7251 |
| Intestinimonas | Fos | −0.1048 | 0.4245 | 0.7251 |
| Intestinimonas | Arc | −0.0984 | 0.4495 | 0.7251 |
| Intestinimonas | Creb1 | −0.0942 | 0.4691 | 0.7427 |
| Intestinimonas | Igf1 | −0.0886 | 0.4963 | 0.7609 |
| Lachnospiraceae__A2 | Dlg4 | 0.1935 | 0.1418 | 0.4350 |
| Lachnospiraceae__A2 | Ngf | 0.1486 | 0.2564 | 0.5898 |
| Lachnospiraceae__A2 | Igf1 | 0.0340 | 0.7943 | 0.9672 |
| Lachnospiraceae__A2 | Fos | −0.0045 | 0.9729 | 0.9968 |
| Lachnospiraceae__A2 | Arc | −0.0163 | 0.9007 | 0.9968 |
| Lachnospiraceae__A2 | Creb1 | 0.0022 | 0.9869 | 0.9968 |
| Lachnospiraceae__ASF356 | Igf1 | 0.3757 | 0.0030 | 0.0525 |
| Lachnospiraceae__ASF356 | Ngf | 0.3267 | 0.0112 | 0.1057 |
| Lachnospiraceae__ASF356 | Arc | 0.2782 | 0.0303 | 0.1818 |
| Lachnospiraceae__ASF356 | Creb1 | 0.2645 | 0.0397 | 0.2109 |
| Lachnospiraceae__ASF356 | Dlg4 | 0.2137 | 0.1040 | 0.3880 |
| Lachnospiraceae__ASF356 | Fos | 0.2006 | 0.1242 | 0.4283 |
| Muribaculum | Creb1 | −0.3924 | 0.0019 | 0.0524 |
| Muribaculum | Igf1 | −0.3755 | 0.0030 | 0.0525 |
| Muribaculum | Arc | −0.3482 | 0.0062 | 0.0729 |
| Muribaculum | Ngf | −0.3013 | 0.0197 | 0.1356 |
| Muribaculum | Fos | −0.2347 | 0.0712 | 0.2892 |
| Muribaculum | Dlg4 | −0.1364 | 0.3021 | 0.6413 |
| Prevotellaceae_UCG-001 | Arc | 0.0933 | 0.4736 | 0.7427 |
| Prevotellaceae_UCG-001 | Creb1 | 0.0916 | 0.4817 | 0.7469 |
| Prevotellaceae_UCG-001 | Ngf | 0.0781 | 0.5520 | 0.7934 |
| Prevotellaceae_UCG-001 | Fos | 0.0674 | 0.6083 | 0.8394 |
| Prevotellaceae_UCG-001 | Igf1 | 0.0421 | 0.7465 | 0.9281 |
| Prevotellaceae_UCG-001 | Dlg4 | −0.0037 | 0.9777 | 0.9968 |
| Ruminococcus | Ngf | −0.1091 | 0.4055 | 0.7251 |
| Ruminococcus | Dlg4 | −0.1109 | 0.4022 | 0.7251 |
| Ruminococcus | Igf1 | −0.0764 | 0.5577 | 0.7934 |
| Ruminococcus | Fos | −0.0781 | 0.5523 | 0.7934 |
| Ruminococcus | Creb1 | −0.0621 | 0.6335 | 0.8487 |
| Ruminococcus | Arc | −0.0120 | 0.9271 | 0.9968 |
| **Tannerellaceae__unknown** | **Ngf** | **−0.4142** | **0.0011** | **0.0380** |
| Tannerellaceae__unknown | Fos | −0.3513 | 0.0062 | 0.0729 |
| Tannerellaceae__unknown | Arc | −0.3238 | 0.0112 | 0.1057 |
| Tannerellaceae__unknown | Creb1 | −0.3174 | 0.0130 | 0.1121 |
| Tannerellaceae__unknown | Igf1 | −0.3014 | 0.0186 | 0.1349 |
| Tannerellaceae__unknown | Dlg4 | −0.2195 | 0.0949 | 0.3638 |
| Turicimonas | Igf1 | 0.1489 | 0.2513 | 0.5898 |
| Turicimonas | Creb1 | 0.1400 | 0.2810 | 0.6155 |
| Turicimonas | Ngf | 0.1031 | 0.4319 | 0.7251 |
| Turicimonas | Arc | 0.0708 | 0.5867 | 0.8179 |
| Turicimonas | Dlg4 | 0.0653 | 0.6224 | 0.8421 |
| Turicimonas | Fos | 0.0016 | 0.9907 | 0.9968 |

Supplementary Table 12. Results Spearman correlations between differentially abundant taxa across FMT groups and behavioral tests scores that also showed significant group-level differences. Statistical significance was adjusted for multiple testing using the false discovery rate (FDR) method. Correlations exceeding |r| > 0.4 with FDR-adjusted *p* values < 0.05 were considered significant.

| **Module** | **Score** | **Correlation** | ***p* value** | ***p* adj** |
| --- | --- | --- | --- | --- |
| Agathobaculum | Sniffing test DI | 0.1269 | 0.3256 | 0.6990 |
| Agathobaculum | Running wheel day | 0.0377 | 0.7706 | 0.8977 |
| Alistipes | Running wheel day | −0.0183 | 0.8878 | 0.9466 |
| Alistipes | Sniffing test DI | −0.0050 | 0.9690 | 0.9950 |
| Anaeroplasma | Sniffing test DI | −0.1710 | 0.1838 | 0.5858 |
| Anaeroplasma | Running wheel day | 0.0457 | 0.7240 | 0.8764 |
| Anaerovoracaceae__Family_XIII_UCG-001 | Running wheel day | −0.1030 | 0.4246 | 0.7512 |
| Anaerovoracaceae__Family_XIII_UCG-001 | Sniffing test DI | −0.0939 | 0.4679 | 0.7541 |
| Anaerovoracaceae__unknown | Sniffing test DI | −0.2616 | 0.0400 | 0.3799 |
| Anaerovoracaceae__unknown | Running wheel day | −0.0645 | 0.6174 | 0.8192 |
| Bacteroides | Running wheel day | −0.2306 | 0.0714 | 0.4315 |
| Bacteroides | Sniffing test DI | 0.0011 | 0.9932 | 0.9980 |
| Barnesiella | Running wheel day | −0.1637 | 0.2032 | 0.5858 |
| Barnesiella | Sniffing test DI | 0.0677 | 0.6013 | 0.8192 |
| Butyricimonas | Running wheel day | −0.4116 | 0.0010 | 0.0525 |
| Butyricimonas | Sniffing test DI | 0.1805 | 0.1604 | 0.5858 |
| Candidatus_Saccharimonas | Sniffing test DI | 0.1927 | 0.1335 | 0.5656 |
| Candidatus_Saccharimonas | Running wheel day | −0.1787 | 0.1644 | 0.5858 |
| Clostridia_UCG-014__unknown | Sniffing test DI | 0.1778 | 0.1668 | 0.5858 |
| Clostridia_UCG-014__unknown | Running wheel day | 0.0640 | 0.6201 | 0.8192 |
| Desulfovibrio | Running wheel day | −0.0813 | 0.5291 | 0.7991 |
| Desulfovibrio | Sniffing test DI | 0.0157 | 0.9033 | 0.9568 |
| Eubacterium_coprostanoligenes_group__unknown | Running wheel day | −0.0872 | 0.4994 | 0.7806 |
| Eubacterium_coprostanoligenes_group__unknown | Sniffing test DI | 0.0265 | 0.8383 | 0.9121 |
| Eubacterium_siraeum_group | Sniffing test DI | 0.0952 | 0.4618 | 0.7541 |
| Eubacterium_siraeum_group | Running wheel day | 0.0831 | 0.5197 | 0.7969 |
| Eubacterium_xylanophilum_group | Running wheel day | −0.1242 | 0.3354 | 0.7105 |
| Eubacterium_xylanophilum_group | Sniffing test DI | −0.0982 | 0.4475 | 0.7541 |
| Intestinimonas | Sniffing test DI | 0.2490 | 0.0510 | 0.3799 |
| Intestinimonas | Running wheel day | −0.1518 | 0.2382 | 0.6184 |
| Lachnospiraceae__A2 | Running wheel day | 0.2675 | 0.0359 | 0.3799 |
| Lachnospiraceae__A2 | Sniffing test DI | −0.1000 | 0.4392 | 0.7541 |
| Lachnospiraceae__ASF356 | Running wheel day | 0.3672 | 0.0035 | 0.1413 |
| Lachnospiraceae__ASF356 | Sniffing test DI | −0.2461 | 0.0538 | 0.3799 |
| Muribaculum | Running wheel day | −0.4247 | 0.0006 | 0.0522 |
| Muribaculum | Sniffing test DI | 0.1088 | 0.3998 | 0.7399 |
| Prevotellaceae_UCG-001 | Sniffing test DI | −0.1199 | 0.3533 | 0.7265 |
| Prevotellaceae_UCG-001 | Running wheel day | 0.0939 | 0.4671 | 0.7541 |
| Ruminococcus | Sniffing test DI | 0.1099 | 0.3953 | 0.7399 |
| Ruminococcus | Running wheel day | 0.0025 | 0.9848 | 0.9972 |
| Tannerellaceae__unknown | Running wheel day | −0.4401 | 0.0004 | 0.0522 |
| Tannerellaceae__unknown | Sniffing test DI | 0.1539 | 0.2324 | 0.6133 |
| Turicimonas | Sniffing test DI | −0.1623 | 0.2076 | 0.5864 |
| Turicimonas | Running wheel day | −0.0482 | 0.7094 | 0.8652 |

Supplementary Table 13. Results Spearman correlations between differentially abundant functional modules across FMT groups and host hippocampal gene expression markers that also showed significant group-level differences. Statistical significance was adjusted for multiple testing using the false discovery rate (FDR) method. Correlations exceeding |r| > 0.4 with FDR-adjusted *p* values < 0.05 were considered significant (in bold).

| **Module** | **Gene** | **Correlation** | ***p* value** | ***p* adj.** |
| --- | --- | --- | --- | --- |
| MF0039 (methionine degradation II) | Ngf | 0.2122 | 0.1035 | 0.2389 |
| MF0039 (methionine degradation II) | Arc | 0.1454 | 0.2629 | 0.4929 |
| MF0039 (methionine degradation II) | Dlg4 | 0.0942 | 0.4771 | 0.6326 |
| MF0039 (methionine degradation II) | Fos | 0.0742 | 0.5720 | 0.6355 |
| MF0039 (methionine degradation II) | Creb1 | 0.0779 | 0.5495 | 0.6355 |
| MF0039 (methionine degradation II) | Igf1 | 0.0581 | 0.6559 | 0.7028 |
| **MF0058 (lysine degradation II)** | **Igf1** | **−0.4539** | **0.0003** | **0.0028** |
| **MF0058 (lysine degradation II)** | **Creb1** | **−0.4566** | **0.0003** | **0.0028** |
| MF0058 (lysine degradation II) | Dlg4 | −0.3964 | 0.0020 | 0.0152 |
| MF0058 (lysine degradation II) | Ngf | −0.3402 | 0.0081 | 0.0302 |
| MF0058 (lysine degradation II) | Arc | −0.3356 | 0.0085 | 0.0302 |
| MF0058 (lysine degradation II) | Fos | −0.3015 | 0.0196 | 0.0588 |
| MGB027 (Nitric oxide degradation) | Ngf | 0.1983 | 0.1286 | 0.2755 |
| MGB027 (Nitric oxide degradation) | Creb1 | 0.1369 | 0.2921 | 0.4934 |
| MGB027 (Nitric oxide degradation) | Igf1 | 0.1297 | 0.3182 | 0.5024 |
| MGB027 (Nitric oxide degradation) | Dlg4 | 0.0818 | 0.5372 | 0.6355 |
| MGB027 (Nitric oxide degradation) | Arc | 0.0739 | 0.5706 | 0.6355 |
| MGB027 (Nitric oxide degradation) | Fos | 0.0144 | 0.9126 | 0.9126 |
| **MGB039 (GHB degradation)** | **Ngf** | **−0.4807** | **0.0001** | **0.0028** |
| MGB039 (GHB degradation) | Creb1 | −0.3742 | 0.0031 | 0.0189 |
| MGB039 (GHB degradation) | Igf1 | −0.3328 | 0.0091 | 0.0302 |
| MGB039 (GHB degradation) | Arc | −0.3418 | 0.0073 | 0.0302 |
| MGB039 (GHB degradation) | Dlg4 | −0.2839 | 0.0297 | 0.0741 |
| MGB039 (GHB degradation) | Fos | −0.2842 | 0.0281 | 0.0741 |
| MGB041 (Menaquinone synthesis) | Fos | 0.1496 | 0.2532 | 0.4929 |
| MGB041 (Menaquinone synthesis) | Igf1 | −0.1357 | 0.2961 | 0.4934 |
| MGB041 (Menaquinone synthesis) | Ngf | 0.1212 | 0.3553 | 0.5330 |
| MGB041 (Menaquinone synthesis) | Dlg4 | 0.1052 | 0.4268 | 0.6097 |
| MGB041 (Menaquinone synthesis) | Creb1 | −0.0909 | 0.4850 | 0.6326 |
| MGB041 (Menaquinone synthesis) | Arc | 0.0206 | 0.8747 | 0.9049 |

Supplementary Table 14. Results Spearman correlations between differentially abundant functional modules across FMT groups and behavioral tests scores that also showed significant group-level differences. Statistical significance was adjusted for multiple testing using the false discovery rate (FDR) method. Correlations exceeding |r| > 0.4 with FDR-adjusted *p* values < 0.05 were considered significant.

| **Module** | **Score** | **Correlation** | ***p* value** | ***p* adj.** |
| --- | --- | --- | --- | --- |
| MF0039 (methionine degradation II) | Running wheel day | 0.2406 | 0.0598 | 0.3488 |
| MF0039 (methionine degradation II) | Sniffing test DI | −0.0398 | 0.7589 | 0.8049 |
| MF0058 (lysine degradation II) | Sniffing test DI | 0.2879 | 0.0233 | 0.3392 |
| MF0058 (lysine degradation II) | Running wheel day | −0.2128 | 0.0968 | 0.3728 |
| MGB027 (Nitric Oxide degradation) | Sniffing test DI | −0.2208 | 0.0846 | 0.3700 |
| MGB027 (Nitric Oxide degradation) | Running wheel day | 0.0259 | 0.8412 | 0.8412 |
| MGB039 (GHB degradation) | Sniffing test DI | 0.2975 | 0.0188 | 0.3392 |
| MGB039 (GHB degradation) | Running wheel day | −0.1947 | 0.1293 | 0.3728 |
| MGB041 (Menaquinone synthesis) | Running wheel day | −0.1558 | 0.2261 | 0.5276 |
| MGB041 (Menaquinone synthesis) | Sniffing test DI | −0.1581 | 0.2196 | 0.5276 |

Supplementary Figure 1. (A) Top glmmTMB_logit features by contrast (mice). Dot plot of metabolite/substrate-level features colored by metabolic subsystem; point size encodes |logFC|. Contrasts: T2DM vs. control, and AD+T2DM vs. control. (B) Top glmmTMB_logit reaction-level features by contrast (mice), faceted by biochemical subsystem. Point size encodes |logFC| and color denotes signed logFC. Reactions with the largest magnitudes are labeled in-panel. Contrasts: T2DM vs. control, and AD+T2DM vs. control.


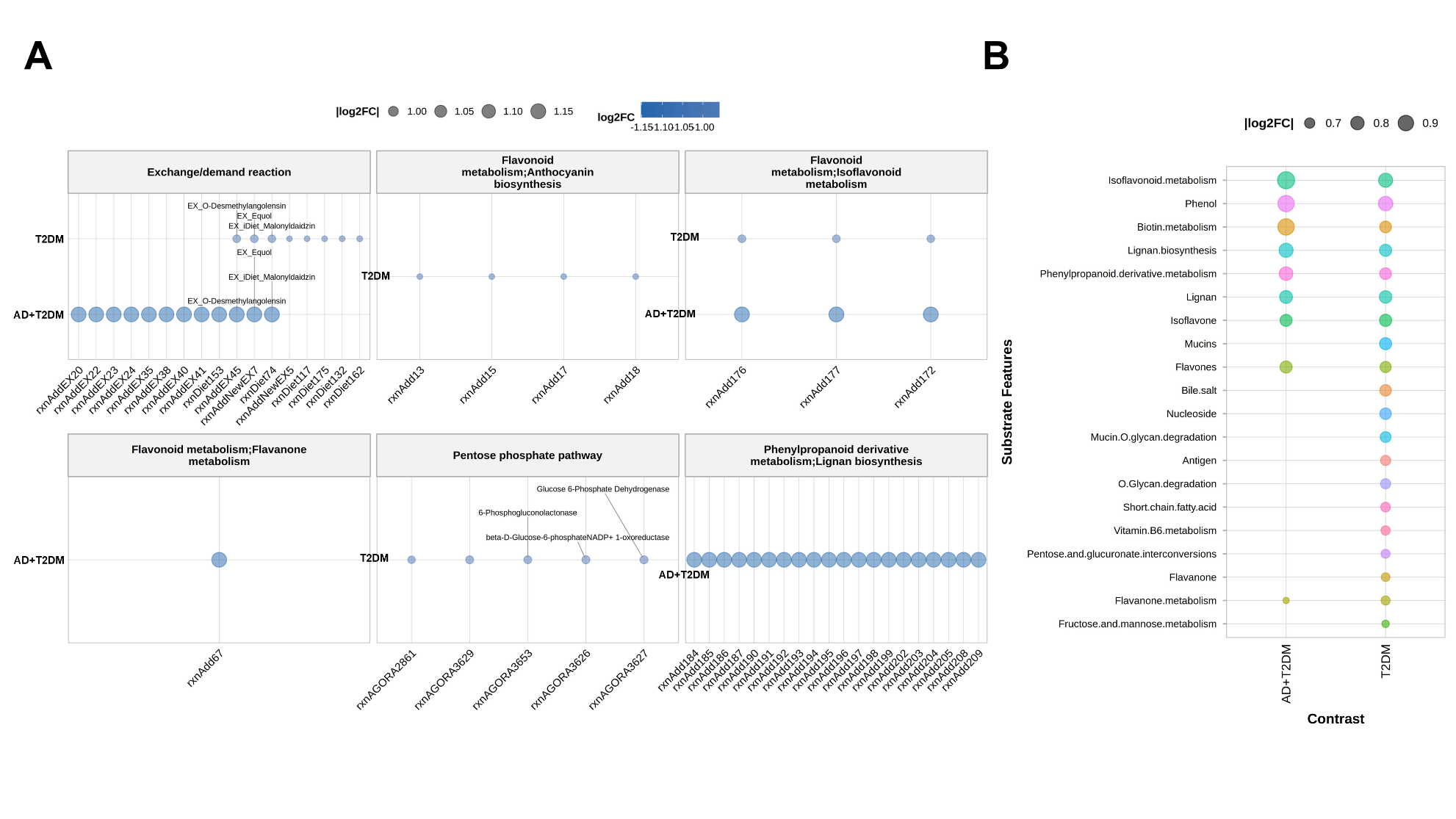


Supplementary Table 15. Top glmmTMB_logit features per contrast (mice) with annotations. [GA] Columns include contrast, feature identifier, subsystem, log fold change (logFC), adjusted *p* value (BH), and any available annotations.

| **Unnamed: 0** | **contrast** | **feature** | **rxnNames** | **subSystems** | **logFC** | **abs_logFC** | ***p* value** | ***p* adj.** |
| --- | --- | --- | --- | --- | --- | --- | --- | --- |
| 30 | AD+T2DM vs. control | rxnAdd172 | new_reaction_172 | Flavonoid metabolism;  Isoflavonoid metabolism | −1.1515 | 1.1515 | 0.0011 | 0.0258 |
| 31 | AD+T2DM vs. control | rxnDiet74 | EX_iDiet_Malonyldaidzin | Exchange/demand reaction | −1.1515 | 1.1515 | 0.0011 | 0.0258 |
| 32 | AD+T2DM vs. control | rxnAdd176 | new_reaction_176 | Flavonoid metabolism;  Isoflavonoid metabolism | −1.1515 | 1.1515 | 0.0011 | 0.0258 |
| 34 | AD+T2DM vs. control | rxnAddEX45 | EX_O-Desmethylangolensin | Exchange/demand reaction | −1.1515 | 1.1515 | 0.0011 | 0.0258 |
| 35 | AD+T2DM vs. control | rxnAddNewEX7 | EX_Equol | Exchange/demand reaction | −1.1515 | 1.1515 | 0.0011 | 0.0258 |
| 33 | AD+T2DM vs. control | rxnAdd177 | new_reaction_177 | Flavonoid metabolism;  Isoflavonoid metabolism | −1.1515 | 1.1515 | 0.0011 | 0.0258 |
| 0 | AD+T2DM vs. control | rxnAdd67 | new_reaction_67 | Flavonoid metabolism;  Flavanone metabolism | −1.1456 | −1.1456 | 0.0030 | 0.0258 |
| 1 | AD+T2DM vs. control | rxnDiet153 | EX_iDiet_Lariciresinol | Exchange/demand reaction | −1.1456 | −1.1456 | 0.0028 | 0.0258 |
| 22 | AD+T2DM vs. control | rxnAddEX20 | EX_Nortrachelogenin | Exchange/demand reaction | −1.1456 | −1.1456 | 0.0028 | 0.0258 |
| 17 | AD+T2DM vs. control | rxnAdd203 | new_reaction_203 | Phenylpropanoid derivative metabolism;  Lignan biosynthesis | −1.1456 | −1.1456 | 0.0028 | 0.0258 |
| 18 | AD+T2DM vs. control | rxnAdd204 | new_reaction_204 | Phenylpropanoid derivative metabolism;  Lignan biosynthesis | −1.1456 | −1.1456 | 0.0028 | 0.0258 |
| 19 | AD+T2DM vs. control | rxnAdd205 | new_reaction_205 | Phenylpropanoid derivative metabolism;  Lignan biosynthesis | −1.1456 | −1.1456 | 0.0028 | 0.0258 |
| 20 | AD+T2DM vs. control | rxnAdd208 | new_reaction_208 | Phenylpropanoid derivative metabolism;  Lignan biosynthesis | −1.1456 | −1.1456 | 0.0028 | 0.0258 |
| 21 | AD+T2DM vs. control | rxnAdd209 | new_reaction_209 | Phenylpropanoid derivative metabolism;  Lignan biosynthesis | −1.1456 | −1.1456 | 0.0028 | 0.0258 |
| 25 | AD+T2DM vs. control | rxnAddEX24 | EX_Trachelogenin | Exchange/demand reaction | −1.1456 | −1.1456 | 0.0028 | 0.0258 |
| 23 | AD+T2DM vs. control | rxnAddEX22 | EX_Sesamin | Exchange/demand reaction | −1.1456 | −1.1456 | 0.0028 | 0.0258 |
| 24 | AD+T2DM vs. control | rxnAddEX23 | EX_Sesamolinol | Exchange/demand reaction | −1.1456 | −1.1456 | 0.0028 | 0.0258 |
| 16 | AD+T2DM vs. control | rxnAdd202 | new_reaction_202 | Phenylpropanoid derivative metabolism;  Lignan biosynthesis | −1.1456 | −1.1456 | 0.0028 | 0.0258 |
| 27 | AD+T2DM vs. control | rxnAddEX38 | EX_ligstroside | Exchange/demand reaction | −1.1456 | −1.1456 | 0.0028 | 0.0258 |
| 28 | AD+T2DM vs. control | rxnAddEX40 | EX_Enterodiol | Exchange/demand reaction | −1.1456 | −1.1456 | 0.0028 | 0.0258 |
| 29 | AD+T2DM vs. control | rxnAddEX41 | EX_Enterolactone | Exchange/demand reaction | −1.1456 | −1.1456 | 0.0028 | 0.0258 |
| 26 | AD+T2DM vs. control | rxnAddEX35 | EX_(+)-episesamin | Exchange/demand reaction | −1.1456 | −1.1456 | 0.0028 | 0.0258 |
| 15 | AD+T2DM vs. control | rxnAdd199 | new_reaction_199 | Phenylpropanoid derivative metabolism;  Lignan biosynthesis | −1.1456 | −1.1456 | 0.0028 | 0.0258 |
| 14 | AD+T2DM vs. control | rxnAdd198 | new_reaction_198 | Phenylpropanoid derivative metabolism;  Lignan biosynthesis | −1.1456 | −1.1456 | 0.0028 | 0.0258 |
| 7 | AD+T2DM vs. control | rxnAdd191 | new_reaction_191 | Phenylpropanoid derivative metabolism;  Lignan biosynthesis | −1.1456 | −1.1456 | 0.0028 | 0.0258 |
| 2 | AD+T2DM vs. control | rxnAdd184 | new_reaction_184 | Phenylpropanoid derivative metabolism;  Lignan biosynthesis | −1.1456 | −1.1456 | 0.0028 | 0.0258 |
| 3 | AD+T2DM vs. control | rxnAdd185 | new_reaction_185 | Phenylpropanoid derivative metabolism;  Lignan biosynthesis | −1.1456 | −1.1456 | 0.0028 | 0.0258 |
| 4 | AD+T2DM vs. control | rxnAdd186 | new_reaction_186 | Phenylpropanoid derivative metabolism;  Lignan biosynthesis | −1.1456 | −1.1456 | 0.0028 | 0.0258 |
| 13 | AD+T2DM vs. control | rxnAdd197 | new_reaction_197 | Phenylpropanoid derivative metabolism;  Lignan biosynthesis | −1.1456 | −1.1456 | 0.0028 | 0.0258 |
| 6 | AD+T2DM vs. control | rxnAdd190 | new_reaction_190 | Phenylpropanoid derivative metabolism;  Lignan biosynthesis | −1.1456 | −1.1456 | 0.0028 | 0.0258 |
| 5 | AD+T2DM vs. control | rxnAdd187 | new_reaction_187 | Phenylpropanoid derivative metabolism;  Lignan biosynthesis | −1.1456 | −1.1456 | 0.0028 | 0.0258 |
| 8 | AD+T2DM vs. control | rxnAdd192 | new_reaction_192 | Phenylpropanoid derivative metabolism;  Lignan biosynthesis | −1.1456 | −1.1456 | 0.0028 | 0.0258 |
| 9 | AD+T2DM vs. control | rxnAdd193 | new_reaction_193 | Phenylpropanoid derivative metabolism;  Lignan biosynthesis | −1.1456 | −1.1456 | 0.0028 | 0.0258 |
| 10 | AD+T2DM vs. control | rxnAdd194 | new_reaction_194 | Phenylpropanoid derivative metabolism;  Lignan biosynthesis | −1.1456 | −1.1456 | 0.0028 | 0.0258 |
| 11 | AD+T2DM vs. control | rxnAdd195 | new_reaction_195 | Phenylpropanoid derivative metabolism;  Lignan biosynthesis | −1.1456 | −1.1456 | 0.0028 | 0.0258 |
| 12 | AD+T2DM vs. control | rxnAdd196 | new_reaction_196 | Phenylpropanoid derivative metabolism;  Lignan biosynthesis | −1.1456 | −1.1456 | 0.0028 | 0.0258 |
| 56 | AD+T2DM vs. control | S153...Isoflavonoid.metabolism |  | 3...Isoflavonoid.metabolism | −0.9935 | 0.9935 | 0.0012 | 0.0320 |
| 51 | T2DM vs. control | rxnAGORA3626 | beta-D-Glucose-6-phosphateNADP+ 1-oxoreductase | Pentose phosphate pathway | −0.9735 | 0.9735 | 0.0009 | 0.0069 |
| 52 | T2DM vs. control | rxnAGORA3627 | Glucose 6-Phosphate Dehydrogenase | Pentose phosphate pathway | −0.9735 | 0.9735 | 0.0009 | 0.0069 |
| 53 | T2DM vs. control | rxnAGORA3653 | 6-Phosphogluconolactonase | Pentose phosphate pathway | −0.9726 | 0.9726 | 0.0009 | 0.0069 |
| 54 | T2DM vs. control | rxnAGORA3629 | Phosphogluconate Dehydrogenase | Pentose phosphate pathway | −0.9725 | 0.9725 | 0.0007 | 0.0069 |
| 41 | T2DM vs. control | rxnAddNewEX7 | EX_Equol | Exchange/demand reaction | −0.9705 | 0.9705 | 0.0013 | 0.0069 |
| 40 | T2DM vs. control | rxnAddEX45 | EX_O-Desmethylangolensin | Exchange/demand reaction | −0.9705 | 0.9705 | 0.0013 | 0.0069 |
| 39 | T2DM vs. control | rxnAdd177 | new_reaction_177 | Flavonoid metabolism;Isoflavonoid metabolism | −0.9705 | 0.9705 | 0.0013 | 0.0069 |
| 38 | T2DM vs. control | rxnAdd176 | new_reaction_176 | Flavonoid metabolism;Isoflavonoid metabolism | −0.9705 | 0.9705 | 0.0013 | 0.0069 |
| 37 | T2DM vs. control | rxnDiet74 | EX_iDiet_Malonyldaidzin | Exchange/demand reaction | −0.9687 | 0.9687 | 0.0013 | 0.0069 |
| 36 | T2DM vs. control | rxnAdd172 | new_reaction_172 | Flavonoid metabolism;Isoflavonoid metabolism | −0.9687 | 0.9687 | 0.0013 | 0.0069 |
| 55 | T2DM vs. control | rxnAGORA2861 | phosphogluconate dehydrogenase, NAD-dependent | Pentose phosphate pathway | −0.9682 | 0.9682 | 0.0007 | 0.0069 |
| 42 | T2DM vs. control | rxnDiet162 | EX_iDiet_Naringenin | Exchange/demand reaction | −0.9600 | 0.9600 | 0.0015 | 0.0069 |
| 43 | T2DM vs. control | rxnDiet132 | EX_iDiet_Genistein | Exchange/demand reaction | −0.9599 | 0.9599 | 0.0015 | 0.0069 |
| 45 | T2DM vs. control | rxnAdd13 | new_reaction_13 | Flavonoid metabolism;Anthocyanin biosynthesis | −0.9598 | 0.9598 | 0.0015 | 0.0069 |
| 46 | T2DM vs. control | rxnAdd15 | new_reaction_15 | Flavonoid metabolism;Anthocyanin biosynthesis | −0.9598 | 0.9598 | 0.0015 | 0.0069 |
| 47 | T2DM vs. control | rxnAdd17 | new_reaction_17 | Flavonoid metabolism;Anthocyanin biosynthesis | −0.9598 | 0.9598 | 0.0015 | 0.0069 |
| 48 | T2DM vs. control | rxnAdd18 | new_reaction_18 | Flavonoid metabolism;Anthocyanin biosynthesis | −0.9598 | 0.9598 | 0.0015 | 0.0069 |
| 49 | T2DM vs. control | rxnDiet175 | EX_iDiet_Peonidin | Exchange/demand reaction | −0.9598 | 0.9598 | 0.0015 | 0.0069 |
| 44 | T2DM vs. control | rxnDiet117 | EX_iDiet_Dihydrocaffeic acid | Exchange/demand reaction | −0.9598 | 0.9598 | 0.0016 | 0.0069 |
| 50 | T2DM vs. control | rxnAddNewEX5 | EX_Phloroglucinol | Exchange/demand reaction | −0.9597 | 0.9597 | 0.0015 | 0.0069 |
| 58 | AD+T2DM vs. control | S79...Biotin.metabolism |  | ...Biotin.metabolism | −0.9509 | 0.9509 | 0.0041 | 0.0320 |
| 57 | AD+T2DM vs. control | S80...Phenol |  | ...Phenol | −0.9491 | 0.9491 | 0.0025 | 0.0320 |
| 67 | T2DM vs. control | S80...Phenol |  | ...Phenol | −0.8484 | 0.8484 | 0.0017 | 0.0084 |
